# Epidemiological Patterns of Cannabis- and Substance- Related Congenital Uronephrological Anomalies in Europe: Geospatiotemporal and Causal Inferential Study

**DOI:** 10.3390/ijerph192113769

**Published:** 2022-10-23

**Authors:** Albert Stuart Reece, Gary Kenneth Hulse

**Affiliations:** 1 Division of Psychiatry, University of Western Australia, Crawley, WA 6009, Australia; 2 School of Medical and Health Sciences, Edith Cowan University, Joondalup, WA 6027, Australia

**Keywords:** tobacco, alcohol, cannabis, cannabinoid, cancer, cancerogenesis, mutagenesis, oncogenesis, genotoxicity, epigenotoxicity, transgenerational inheritance

## Abstract

Introduction. Recent reports linking prenatal and community cannabis exposure to elevated uronephrological congenital anomaly (UCA) rates (UCAR’s) raise the question of its European epidemiology given recent increases in community cannabinoid penetration there. Methods. UCAR data from Eurocat. Drug use data from European Monitoring Centre for Drugs and Drug Addiction. Income from World bank. Results. UCAR increased across Spain, Netherlands, Poland and France. UCAR’s and cannabis resin THC increased simultaneously in France, Spain, Netherlands and Bulgaria. At bivariate analysis all UCA’s were related to cannabis herb and resin THC concentrations. All UCAR’s were bivariately related to cannabis metrics ordered by median minimum E-value (mEV) as hypospadias > multicystic renal disease > bilateral renal agenesis > UCA’s > hydronephrosis > posterior urethral valve > bladder exstrophy/epispadias. At inverse probability weighted multivariable analysis terms including cannabis were significant for the following series of anomalies: UCA’s, multicystic renal disease, bilateral renal agenesis, hydronephrosis, congenital posterior urethral valves from P = 1.91 × 10^−5^, 2.61 × 10^−8^, 4.60 × 10^−15^, 4.60 × 10^−15^ and 2.66 × 10^−10^. At geospatial analysis the same series of UCA’s were significantly related to cannabis from P = 7.84 × 10^−15^, 7.72 × 10^−5^, 0.0023, 6.95 × 10^−5^, and 8.82 × 10^−5^. 45/51 (88.2%) of E-value estimates and 31/51 (60.8%) of mEV’s >9. Conclusion. Analysis confirms a close relationship between cannabis metrics and all seven UCA’s and fulfill formal criteria for quantitative causal inference. Given the exponential cannabinoid genotoxicity dose–response relationship results provide a powerful stimulus to constrain community cannabinoid exposure including protection of the food chain to preserve the genome and epigenome of coming generations.

## 1. Introduction

### 1.1. Background

Recent reports demonstrate increasing concern at the genotoxic activities of cannabinoids expressed as congenital anomalies, cancer and aging [1,2,3,4,5,6,7,8,9,10,11,12,13,14,15,16,17,18,19,20,21,22,23]. Recent epidemiological teratological reports have causally linked cannabis exposure with cardiovascular, chromosomal, central nervous system, limb, gastrointestinal, orofacial and body wall congenital anomalies [6,7,8,12,15,18,24,25,26,27,28,29,30,31]. A prominent part of cannabis teratology is uronephrological (urinary tract and renal) congenital anomalies (UCA’s) which have featured in reports of elevated rates of renal agenesis in Colorado [13], obstructive genitourinary defect in Hawaii [19], congenital posterior urethral valve, obstructive genitourinary defect, renal agenesis/hypoplasia, hypospadias and epispadias in USA [7] and hypospadias in Australia [8]. It was therefore of interest to investigate contemporary trends of this group of anomalies in Europe particularly given the rising triple convergence of prevalence of daily use, intensity of daily use and rising cannabinoid potency in widely available products in many countries across the European continent in the recent decade [20,32].

### 1.2. Cannabinoid Genotoxicity

Cannabinoid-related teratogenesis forms a subset of the large subject of cannabinoid genotoxicity which also includes cannabinoid related carcinogenesis [1,2,3,4,5,23] and cannabinoid induced acceleration of aging [7,11,12,23,33,34,35,36]. It is important to keep these broader issues in mind when considering the present investigation.

### 1.3. Genotoxic Mechanisms

Cannabinoid genotoxicity has long been known to occur by many mechanisms including gross abnormalities of sperm morphology [37,38], disruptions of normal oocyte division [39], many changes to oviduct and spermatogenic niche physiology [40,41,42], reduction in histone-protamine transfer during sperm DNA packing [42], chromosomal breaks [37,43,44,45], single- and double- stranded DNA breaks [43], mutagenic oxidation of DNA bases [43], chromosomal end-to-end fusions and translocations [37], likely breakage-fusion-bridge cycles in testicular oncogenesis [46,47], changes in DNA methylation patterns in sperm which are transmissible to following generations [48,49,50,51,52,53,54,55], reductions in histone synthesis [56,57,58,59,60] and reductions in post-translational modifications of histones [59,60,61] which are intergenerationally transmissible [62]. All of these mechanisms have been importantly implicated in cannabis related carcinogenesis [1,2,3,4,5,9,11,26,29,63]. Similarly these and other mechanisms have been implicated in the cannabis induced impairment of male fertility [64].

### 1.4. Exponential Dose-Response Effect Curve

It is important to appreciate that the genotoxic action of cannabinoids is strongly exponential with effects rising dramatically in the higher dose range. This has been noted both for the direct DNA mutagenicity [43,65,66,67,68,69,70,71,72] and for the mitochondrial metabolic reactions which underpin genomic and epigenomic reactions and provide substrates and energy to the genetic and epigenetic machinery [67,73,74,75,76,77,78]. This implies in turn that the rising triple confluence of prevalence–intensity-potency mentioned above can relatively abruptly place communities into a zone of high genotoxic risk result in a relatively sudden appearance of major genotoxic outcomes [20,32]. This likely accounts for adverse experiences such as recent French and German outbreaks of limblessness at up to sixty times the background rate in areas where many hectares are sown to cannabis [79,80,81,82]. Interestingly not only are babies being born without limbs but so too are the calves suggesting cannabinoid exposure via the food chain [79,80,81]. However, none of these effects are happening in nearby Switzerland where cannabinoids are not permitted in foodstuffs.

### 1.5. Lessons from VACTERL Syndrome

Interestingly a companion paper to this one has shown that the VACTERL syndrome (Vertebral, anorectal, cardiac, tracheo-esophageal fistulae/esophageal atresia, renal and limb anomalies) [83] was strongly and causally linked with European cannabinoid exposure [15,84]. Since renal anomalies are part of the VACTERL syndrome this finding also imputes renal abnormalities in the overall pattern of uronephrological congenital anomalies (UCA’s).

### 1.6. Study Questions

For these reasons it was considered important to assess UCA rates (UCAR’s) in response to increased cannabinoid exposure across many European countries, particularly as UCA’s appear to be a relatively sensitive mutagenic index of cannabinoid teratology. It was also considered important that this assessment be undertaken in a bivariate and multivariable context within a causal inferential framework, and in a spatiotemporal context which formally takes account of the native space-time environment in which this data was generated.

## 2. Methods

### 2.1. Data

The data on congenital anomaly rates was downloaded from the European Network of Population-Based Registries for the Epidemiological Surveillance of Congenital Anomalies (EUROCAT) website [85]. Annual data for all available anomalies was downloaded from both the summary and the detailed files for 14 nations for each individual year. The metric of interest was the total congenital anomaly rate which includes live births, stillbirths and cases where early termination for anomaly was practised. The total rate therefore represents the rate net of all the different birth exigencies to which pregnancies are subject. Nations were selected based upon most of their congenital anomaly data being available across most of the period 2010–2019. Data on national tobacco (percent daily tobacco use prevalence) and alcohol (litres of pure alcohol consumed per capita annually) consumption were obtained from the World Health Organization website [86]. The website of the European Monitoring Centre for Drugs and Drug Addiction (EMCDDA) [87] was used to source data on drug use relating to cannabis, amphetamines and cocaine. The major metric of cannabis exposure was last month cannabis use data. This was supplemented by data on the tetrahydrocannabinol (THC) content of cannabis herb and resin provided in recent published reports [32] and by data on daily cannabis use which was also available from EMCDDA and was collated in recent Public Health reports [32]. Data on median household income data (in $USD) was derived from the World Bank sources [88]. 

### 2.2. National Assignment

Nations were grouped into either low and/or falling daily cannabis use or high and/or rising daily cannabis use based on a recent European epidemiological study (see Supplementary Figure S4 of reference [32]). In this way France, Germany, Italy, Belgium, Croatia, Norway, Portugal, Netherlands and Spain were categorized as nations experiencing increasing daily use. Finland, Hungary, Poland, Bulgaria and Sweden were nations which were experiencing low or falling levels of daily cannabis use.

### 2.3. Derived Data

Because several metrics of cannabis exposure were available it was possible to combine and group these in various ways. Thus last month cannabis use prevalence data was multiplied by the THC content of cannabis resin and herb to derive compound metrics from their product. These metrics were again multiplied by imputed daily cannabis use prevalence rates to derive more complex compound metrics for both cannabis resin and herb.

### 2.4. Data Imputation

Linear interpolation across years was used to complete missing data. This was especially so for daily cannabis use. For these 14 nations 59 data points on daily cannabis use were available from EMCDDA across this period. Linear interpolation was able to expand this dataset out to 129 datapoints (further details are provided within Results Section). There was no available data on cannabis resin THC concentration for Sweden. It was noted however that the resin: herb THC concentration was virtually in nearby Norway at 17.7. This ratio was therefore applied to the Swedish cannabis herb THC concentration data to derive estimates of the Swedish cannabis resin THC concentration. In a similar manner data for the cannabis resin THC concentration in Poland were not available from EMCDDA. The resin to herb THC concentration ratio of nearby Germany across time was used to estimate the resin THC content in Poland from the known Polish herb THC concentrations. Since geospatial analytical techniques do not tolerate missing data the dataset was completed by interpolation by the last observation carried forward or backwards for Croatia in 2018 and 2019 and Netherlands in 2010. Multiple imputation methods could not be used for these analyses as multiple imputation techniques have not been applied to panel or spatial multivariable regression methods. 

### 2.5. Statistics

R Studio version 1.4.1717 based on R version 4.1.1 from the Comprehensive R Archive Network and the R Foundation for Statistical Computing was used for data processing [89]. The analysis was performed in December 2021. Data manipulation was performed using dplyr from the R Core team tidyverse [90]. Data were log transformed where appropriate as guided by the Shapiro–Wilks test to improve compliance with normality assumptions. ggplot2 from tidyverse was used for constructing graphs. Both ggplot2 and sf (simple features) [91] were used for drawing maps using both custom colour palettes and palettes provided in the viridis and viridisLite packages [92]. Bivariate maps were drawn with package colorplaner by William Murphy [93]. Illustrations are all original and have not been previously published. Linear regression was conducted in R Base. R package nlme was used for mixed effects regression [94]. Model reduction was by the classical technique of serial deletion of the least significant term to yield a final reduced model which is the model presented in all multivariable models. Combined techniques from R packages purrr and broom were used to process multiple linear models in a single pass virtually simultaneously [90,95,96]. The overall effect of covariates particularly of interactive multivariable models may be quantified and is denoted as the marginal effect. The R package margins was used to calculate the overall marginal effect [97].

### 2.6. Covariate Selection

With numerous different metrics of cannabis exposure available it was unclear which was the most appropriate set of covariates to select for each individual model. Use of excessive cannabis related covariates could lead to issues of collinearity, consumption of excessive degrees of freedom and a concomitant limitation of interactions which could be considered, or model mis-specification or imbalancing more generally. 

The presence of multiple different metrics for cannabis consumption and exposure created a problem for analysis as it was not clear which was the most appropriate metric to employ for any particular model. Indiscriminate use of excessive covariates in a multivariable model would unnecessarily consume degrees of freedom and thereby restrict ability to assess interactions. Random forest regression using the R package ranger [98] with variable importance being formally assessed via the R package vip (variable importance plot) [99] was used to formally address this issue of variable selection. The most predictive of the covariates identified by this process were then entered into the regression modelling equations. Applicable tables from these analysis are presented below in the Results Section. 

### 2.7. Panel and Geospatial Analysis

The R package plm [100] was utilized to conduct panel analysis across both space and time simultaneously using the “twoways” effect. The spatial weights matrix for temporospatial modelling was calculated using the edge and corner “queen” relationships using R package spdep (spatial dependency) [101]. The spatial panel random effects maximum likelihood (spreml) function from the package spml which allows detailed modelling and correction of model error structures [102,103] was used for geospatial modelling. Such models can produce up to four descriptive model coefficients which are can be used to verify the most appropriate error structure for the model. These coefficients are psi the serial correlation effect, rho the spatial coefficient, phi the random error effect and theta the spatial autocorrelation coefficient. The most appropriate error structure was chosen for each series of spatial models taking care to preserve the model error specification across related models. The appropriate error structure was determined by the backwards method from the full general model to the most specific model by the method which has previously been published [104]. Temporal lagging by one or two years was employed as indicated in both panel and geospatial models.

### 2.8. Causal Inference

This analysis deployed the formal tools of causal inference. Inverse probability weighting (ipw) is the analytical technique of choice to convert a purely observational study into a pseudo-randomised study from which it is entirely appropriate to draw causal inferences [105]. Inverse probability weighting was employed in all multivariable panel models presented. Inverse probability weights were calculated via the R package ipw. Similarly E-values (expected values) quantify the correlation required of some hypothetical unmeasured confounder covariate with both the exposure of concern and the outcome of interest in order to explain away some relationship which appears to be apparently causal in nature [106,107,108]. It therefore provides a quantitative measure of the robustness of the model to extraneous covariates which have not been accounted for within the measured parameters and is a popular and increasingly published form of sensitivity analysis. E-Values have a measure o uncertainty associated with them which can be quantified as a confidence interval and the 95% lower bound of this confidence interval is particularly relevant to situations of increased risk. For these reasons it has been extensively reported herein. E-Value estimates greater than 1.25 to indicate causality [109] and E-values larger than nine are described as being high [110]. The R package EValue was used for the calculation of both E-value estimates and their 95% lower bounds [111]. Both inverse probability weighting and E-values are foundational and pivotal techniques used in formal quantitative causal inferential methods in order to allow causal relationships to be assessed from real world observational studies and to control for the effect of observed and unobserved covariates respectively.

### 2.9. Data Availability

Raw datasets including 3800 lines of computation code in R has been made freely available through the Mendeley data repository at the following URL’s: https://data.mendeley.com/datasets/c6psrbr34j/1 and https://data.mendeley.com/datasets/vd6mt5r5jm/1. 

### 2.10. Ethics

The Human Research Ethics Committee of the University of Western Australia provided ethical approval for the study to be undertaken 24 September 2021 (No. RA/4/20/4724). All methods were carried out in accordance with relevant guidelines and regulations and were concordant with the Declaration of Helsinki. 

## 3. Results

### 3.1. Input Data

Supplementary Table S1 sets out the general overview of the covariates used in this study. As indicated 839 data points were downloaded from the EUROCAT database from the 14 nations shown and applying to the seven CA’s of urinary disorders, bilateral renal agenesis, bladder exstrophy or epispadias, congenital hydronephrosis, multicystic renal dysplasia and posterior urethral valve or prune belly. Covariates for drug exposure including various primary and compound metrics of cannabis exposure are as indicted in the table along with median household income data.

Data on daily drug use by European nation and year was examined and was found to be incomplete as indicated in Supplementary Table S2 with 59 points available. In order to allow this information to be used in comparative studies a further 70 datapoints were added to this data by linear interpolation. This augmented dataset is shown in Supplementary Table S3.

### 3.2. Bivariate Analysis

**Figure 1:**
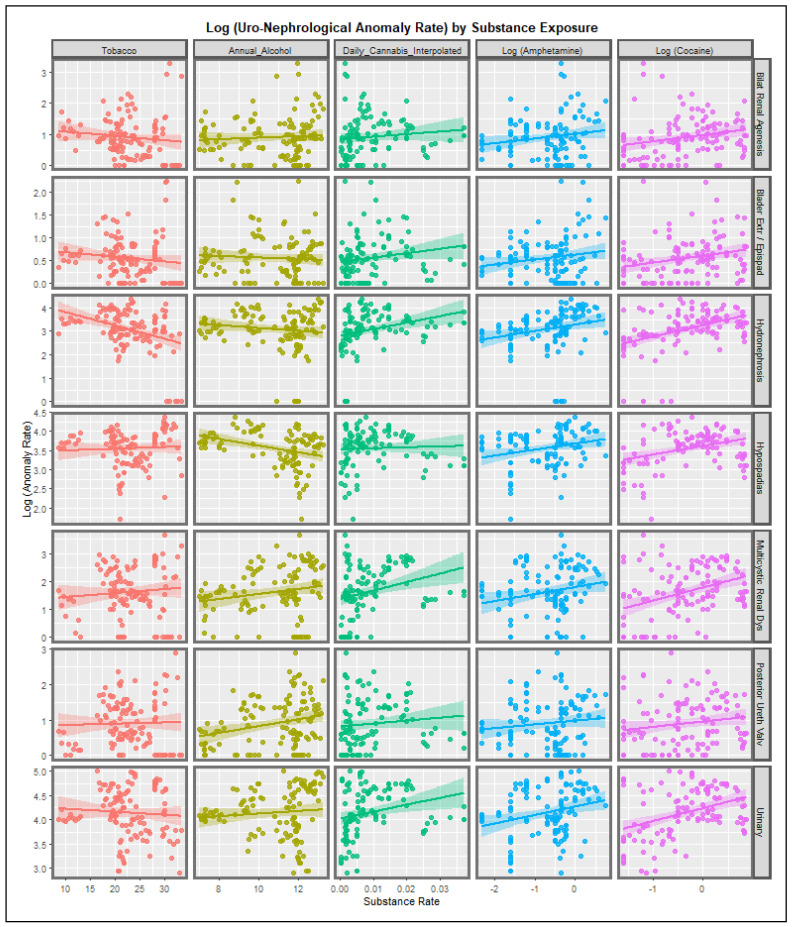
Bivariate plots of selected uronephrological congenital anomalies against various substances.

Figure 1 shows the relationship between the rates of the various UCA’s and the substance tobacco, alcohol, daily cannabis use interpolated, amphetamine and cocaine exposure. It appears that tobacco exposure is not associated with any UCA incidence. There does seem to be a positive relationship between annual alcohol consumption and multicystic renal disease and posterior urethral valve as those regression lines have a positive slope. Amphetamine and cocaine exposure appear to be positively related to all defects mentioned. Daily cannabis interpolated appears to be related to hydronephrosis, multicystic renal disease and urinary anomalies globally.

**Figure 2:**
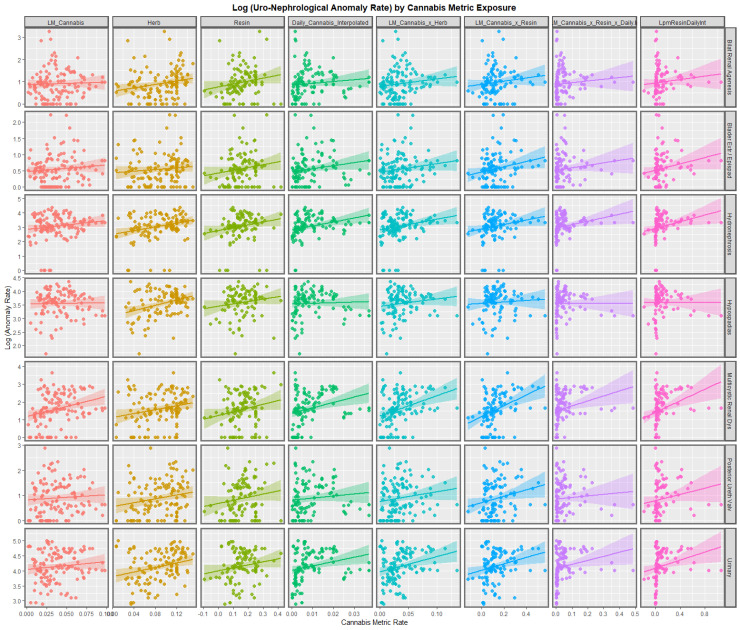
Bivariate plots of selected uronephrological congenital anomalies against various metrics of cannabis exposure.

Figure 2 illustrates the relationship between various cannabis exposure metrics and rates of the various UCA’s. Many of the regression lines appear to be positively up-sloping including those for last month cannabis use, the THC concentration of cannabis herb and resin, and most of the compound cannabis metrics.

**Figure 3:**
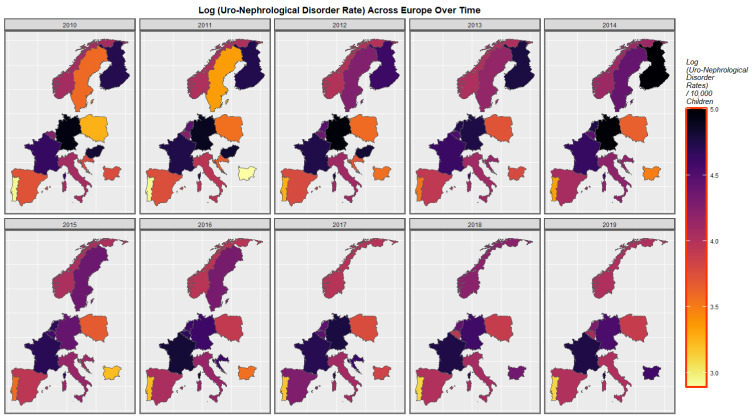
Time sequential map-graph of log (uronephrological anomaly rate) across surveyed European nations over time.

Figure 3 shows the rates of UCA’s across Europe for the past decade. Rates seem to have risen in Spain, Portugal, France, Belgium, Bulgaria, Sweden and Croatia but have declined in Germany.

**Figure 4:**
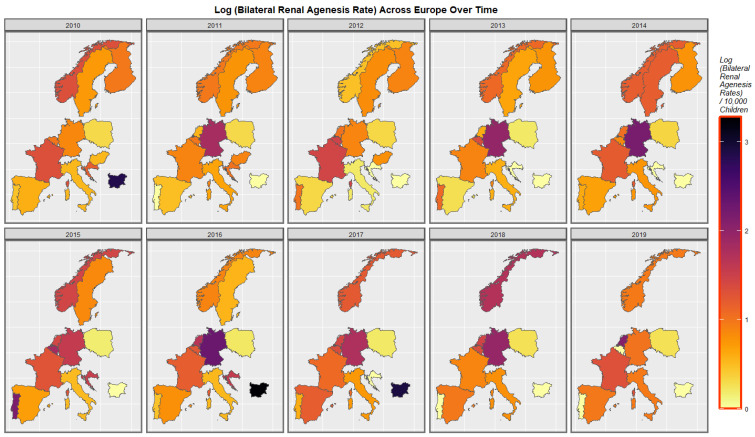
Time sequential map-graph of log (bilateral renal agenesis anomaly rate) across surveyed European nations over time.

Rates of bilateral renal agenesis are shown in Figure 4. The appear to have increased in Spain, Norway, Germany, Netherlands, Belgium and Croatia but reduced in Poland.

**Figure 5:**
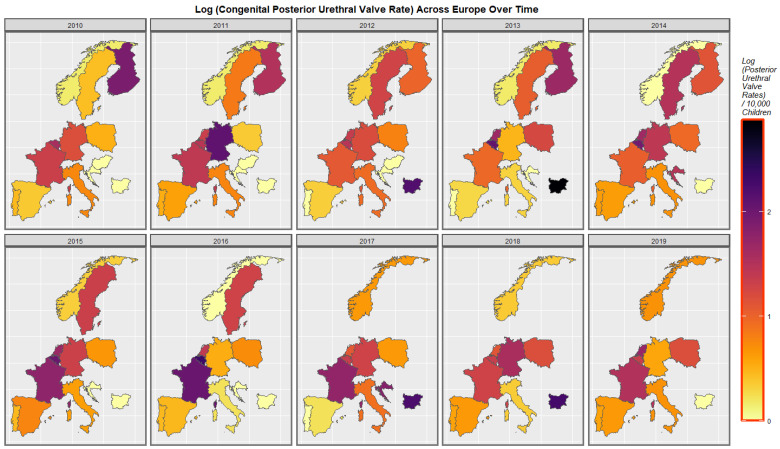
Time sequential map-graph of log (congenital posterior urethral valve rate) across surveyed European nations over time.

Rates of posterior urethral valve are shown in Figure 5. Rates increased in Spain, Portugal, Poland, France, Bulgaria and Sweden but reduced in Germany. Rates in the low countries and France fluctuated.

**Figure 6:**
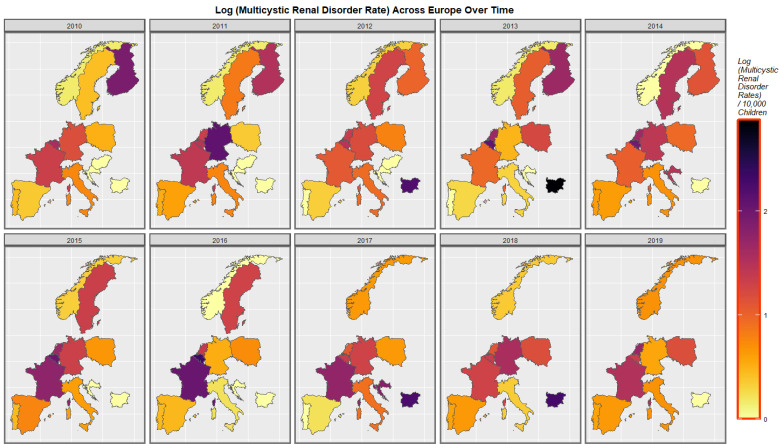
Time sequential map-graph of log (Multicystic renal disease rate) across surveyed European nations over time.

Rates of multicystic renal disease are shown in Figure 6. Rates increased in France, Bulgaria, Norway, Poland, Netherlands, Belgium, Croatia and Sweden and fluctuated in Germany.

**Figure 7:**
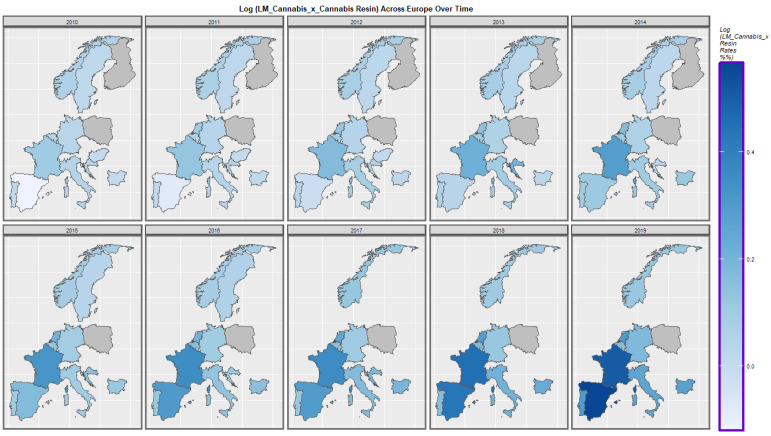
Time sequential map-graph of last month cannabis use: cannabis resin THC concentration across surveyed European nations over time.

Figure 7 shows the rate of the compound metric last month cannabis use: cannabis resin THC concentration. It has increased across the continent but the rises in Spain, Portugal, Netherland and France were most accentuated.

#### 3.2.1. Bivariate Graphs

**Figure 8:**
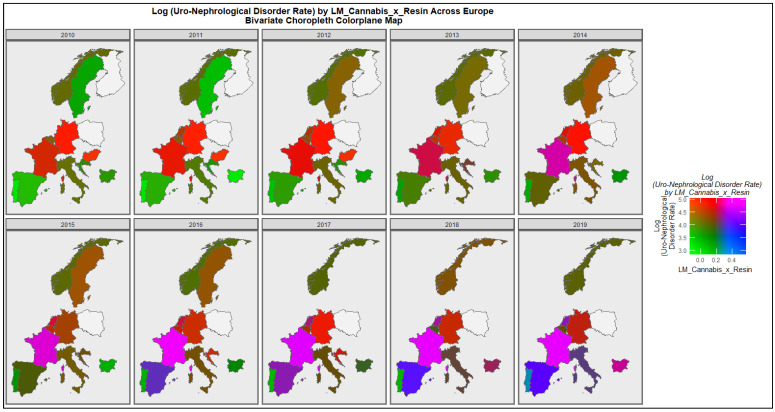
Colorplane bivariate time sequential map-graph of log (uronephrological congenital anomaly rate) by last month cannabis use: cannabis resin THC concentration across surveyed European nations over time.

Figure 8 is a bivariate plot of the co-distribution of uronephrolgical congenital disorders and last month cannabis use: cannabis resin THC concentration across Europe over the decade. One reads the plot by noting the areas which turn purple or crimson which signify a high distribution of both covariates. The significance of the other colours is indicated in the colorplane key. The series of graphs makes clear the co-emergence of high rates of both uronephrolgical disease and the compound cannabis use metric in France, Netherlands and Bulgaria across the decade.

**Figure 9:**
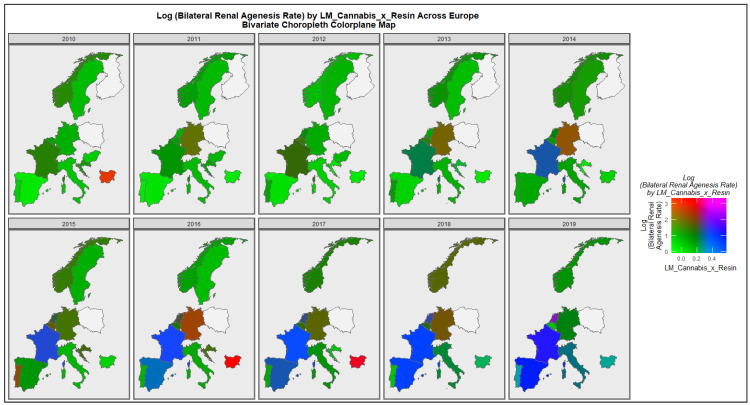
Colorplane bivariate time sequential map-graph of log (bilateral renal agenesis congenital anomaly rate) by last month cannabis use: cannabis resin THC concentration across surveyed European nations over time.

Figure 9 is a similar bivariate series of maps showing the co-emergence of high rates of bilateral renal agenesis and the compound cannabis exposure metric in Netherlands.

**Figure 10:**
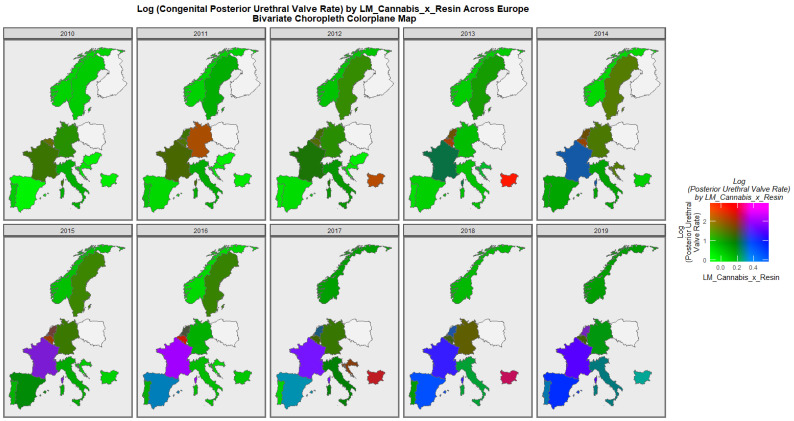
Colorplane bivariate time sequential map-graph of log (posterior urethral valve congenital anomaly rate) by last month cannabis use: cannabis resin THC concentration across surveyed European nations over time.

Figure 10 plays the same role for rates of posterior urethral valve. The co-emergence of high rates in France and Netherlands is made plain by this graphic.

**Figure 11:**
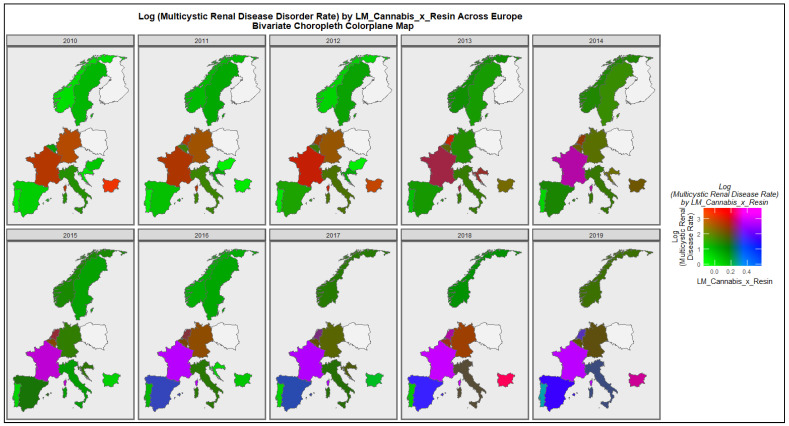
Colorplane bivariate time sequential map-graph of log (multicystic renal disease congenital anomaly rate) by last month cannabis use: cannabis resin THC concentration across surveyed European nations over time.

Figure 11 performs a similar function for multicystic renal disease. The co-emergence of high rates in France, Netherlands and Bulgaria is shown.

#### 3.2.2. Bivariate Overview

**Figure 12:**
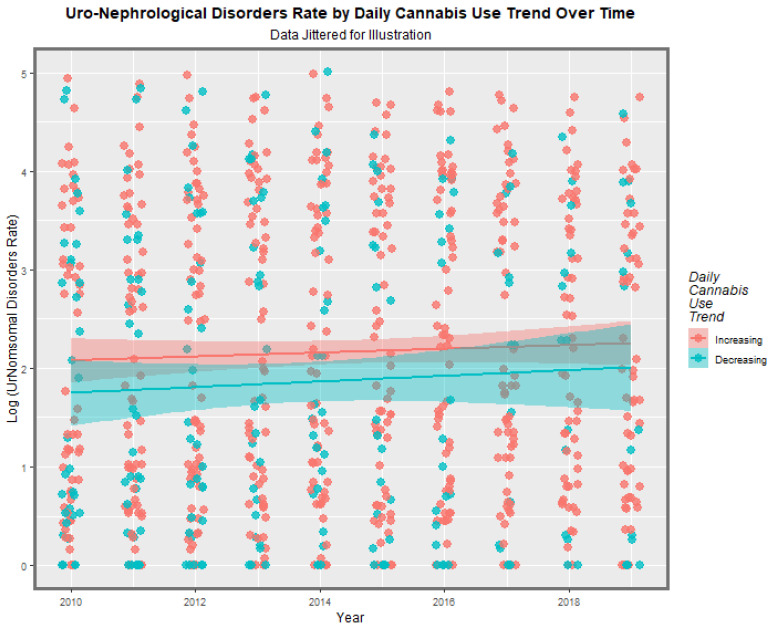
Overall rate of log (uronephrolgical congenital anomaly rate) against time by rising of falling daily cannabis use rates (see Methods section for country classification).

Nations were grouped into those where daily cannabis use was increasing or high and those where it is not based on recent epidemiological reports [32]. When all CA’s are groups together the appearances seen in Figure 12 are shown. Nations where daily use is increasing are shown to have higher rates of UCA’s at linear regression (β-est. = 0.3121, t = 2.687, *p* = 0.0073; model Adj.R.Squ. = 0.074, F = 75.22, df = 12, 837, *p* = 0074). The E-value estimate for this effect is 1.71 with a 95% lower bound on the confidence interval (mEV) of 1.29 which exceeds the quoted threshold fror causality of 1.25 [109]

**Figure 13:**
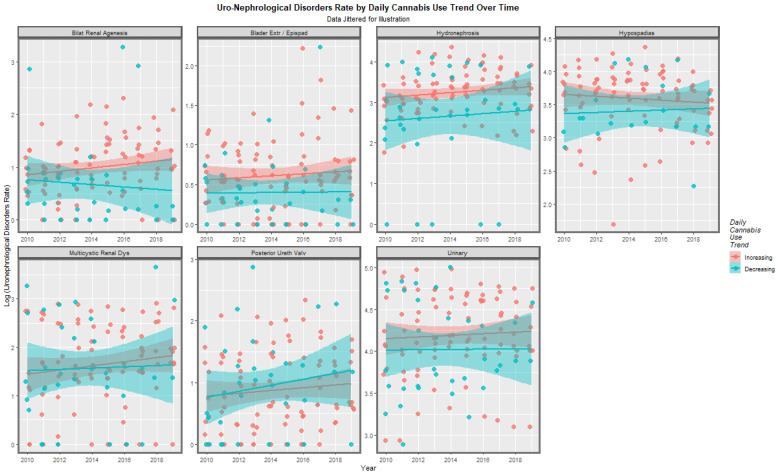
Rate of log (uronephrological congenital anomaly rate) against time by rising of falling daily cannabis use rates by congenital anomaly.

When this comparison is done in an anomaly-specific manner the appearances shown in Figure 13 are seen. When these differences are studied formally by mixed effects regression the rate in the countries with increasing daily use is significantly higher than the remainder (β-est. = 0.2048, t = 3.943, *p* = 8.73 × 10^−5^; model AIC = 1755.781, Log.Lik = −872.891). This effect is associated with an E-value estimate of 1.97 and mEV = 1.57.

#### 3.2.3. Bivariate Linear Regressions

The regression lines shown graphically in Figure 1 and Figure 2 may be studied by multiple bivariate linear regression models simultaneously using the purrr-broom workflow in R. when this is performed 85 regression models are derived which are listed in Supplementary Table S2 in descending order of mEV. Interestingly daily cannabis use appears at the top of this list of models.

From this list those which have positive regression coefficients and significant *p*-values may be extracted. 37 such models are retained in this way which are listed in Table 1.

### 3.3. Covariate Selection

Having identified so many bivariate relationships of interest the next question relates to how these different covariates might perform in a multivariable context. However, given that there are 13 substance exposure covariates it is not entirely obvious which might be the best combination of covariates to use in anomaly-specific regression equations.

This issue was addressed formally by the use of random Forest regression in tandem with variable importance assessment using the R packages ranger and vip. When this was done for the overall UCA’s and for multicystic renal disease, bilateral renal agenesis, hydronephrosis and posterior urethral valve the variable importance tables shown in Supplementary Tables S3–S7 were derived. 

### 3.4. Multivariable Regressions

#### 3.4.1. IPW Panel Regression

These covariates were then used to define multivariable inverse probability weighted panel regression models for a series of multivariable models relating to the five congenital uronephrological disorders of interest. The inverse probability weighting is of considerable importance and allows the analysis to move from that of an observational study only to a pseudo-randomized milieu from which is it appropriate to draw causal inferences.

Supplementary Table S8 describes three such final inverse probability weighted panel regression models for uronephrological disease as a whole. Additive, interactive and temporally lagged models are shown. In each one cannabis metrics are significant in final models and survive model reduction, have positive regression coefficients and are highly statistically significant.

This pattern is continued across the other anomalies, namely multicystic renal dysplasia, bilateral renal agenesis, hydronephrosis and posterior urethral valve syndrome as shown in Supplementary Tables S9–S12. 

This result is important because it means directly that in this set of congenital anomalies the strong trends identified at bivariate regression persist after multivariable adjustment in an inverse probability weighted and therefore casual paradigm.

#### 3.4.2. Geospatial Regression

The next issue to consider was whether these relationships would continue when considered formally in their native space-time context in which major methodological concerns such as random errors, serial correlation, spatial correlation and spatial autocorrelation would be properly accounted for and managed.

Supplementary Figure S1 presents maps of the initial, edited and final geospatial links between countries which were derived and used as the basis for the sparse spatial weights matrix used in the spatial regressions.

Table 2 presents additive, interactive and lagged geospatial models for uronephrological disorders broadly. The pattern identified earlier in panel regression is continued with some cannabis-related terms in each model persisting in the final model after model reduction and maintaining high levels of statistical significance from 7.84 × 10^−15^ in the case of the first additive model. 

This pattern is continued when multicystic renal disease is considered as shown in Table 3. In each model the effect of cannabis-related covariates is overwhelmingly in the positive direction.

Similar results are also seen when bilateral renal agenesis is considered in interactive and lagged models as shown in Table 4.

When hydronephrosis is considered the pattern is continued in additive, interactive and models lagged to one year, but the signal fades by two years of temporal lagging (Table 5).

The pattern is continued and confirmed when posterior urethral valve syndromes are considered as shown in Table 6. In each case terms including cannabis metrics persist after adjustment in final models, have positive regression coefficients and are statistically significant. 

### 3.5. Causal Inference

#### E-Values

E-values may be extracted from each of these regression models. Table 7 does this for panel models and Table 8 does this for spatial models.

Both lists are combined in Table 9 where 51 such E-value pairs are listed in descending order of mEV. The prominence of daily cannabis use interpolated at the head of the table is noted as is the high level of statistical significance of the E-values returned. 

**Table 1:** Positive Significant Slopes from Bivariate Linear Regressions.

Anomaly	Substance	Mean Anomaly Rate	Estimate	Std.Error	Sigma	t_statistic	*p*-Value	E-Value Estimate	E-Value Lower Bound
Multicystic Renal Dys	Daily.Interpol.	3.4655	30.4447	9.5953	0.9007	3.1729	0.0019	4.57 × 10^13^	2.66 × 10^5^
Hydronephrosis	Daily.Interpol.	13.8455	27.8771	8.7897	0.8250	3.1716	0.0020	4.52 × 10^13^	2.62 × 10^5^
Urinary	Daily.Interpol.	35.4475	14.3684	5.3583	0.5029	2.6815	0.0084	3.90 × 10^11^	2.27 × 10^3^
Multicystic Renal Dys	LMCannabis_Herb	3.4655	11.4522	3.0267	0.8688	3.7837	2.43 × 10^−4^	3.24 × 10^5^	656.0696
Hypospadias	Herb	19.2037	4.9196	1.3727	0.4546	3.5838	5.15 × 10^−4^	3.78 × 10^4^	174.7051
Multicystic Renal Dys	LM_Cannabis	3.4655	11.4477	3.6514	0.8837	3.1352	0.0022	2.63 × 10^5^	167.9388
Urinary	LMCannabis_Herb	35.4475	5.0871	1.7232	0.4947	2.9521	0.0038	2.32 × 10^4^	46.5273
Hydronephrosis	Daily.Interpol.	13.8455	6.5820	2.1094	0.8202	3.1204	0.0023	2.97 × 10^3^	30.0042
Urinary	Herb	35.4475	3.8164	1.2706	0.4941	3.0036	0.0032	2.26 × 10^3^	22.7009
Hydronephrosis	LMCannabis_Herb	13.8455	7.3128	2.8950	0.8310	2.5260	0.0128	6.01 × 10^3^	11.6605
Multicystic Renal Dys	LMCannabis_Resin	3.4655	3.0558	0.7366	0.8820	4.1488	6.81 × 10^−5^	46.3050	10.0602
Multicystic Renal Dys	Herb	3.4655	5.5861	2.3083	0.8976	2.4200	0.0170	575.8297	5.3720
Bilat Renal Agenesis	Herb	1.3617	3.9363	1.6424	0.6386	2.3967	0.0181	545.3091	5.0547
Multicystic Renal Dys	LM.Cannabis_x_Herb.THC_x_Daily.Interpol.	3.4655	2.8187	1.0685	0.9122	2.6379	0.0095	32.7720	3.5552
Posterior Ureth Valv	Herb	1.3062	3.8400	1.7052	0.6630	2.2519	0.0261	388.4327	3.4114
Multicystic Renal Dys	LM.Cannabis_x_Resin.THC_x_Daily.Interpol.	3.4655	1.6437	0.4955	0.9272	3.3171	0.0013	9.5088	3.2871
Hydronephrosis	LM.Cannabis_x_Herb.THC_x_Daily.Interpol.	13.8455	2.4167	0.9825	0.8388	2.4597	0.0154	27.0122	2.8132
Urinary	LMCannabis_Resin	35.4475	1.0733	0.4108	0.4919	2.6129	0.0103	14.0498	2.6800
Hydronephrosis	Cocaine	13.8455	0.4916	0.0962	0.7729	5.1079	1.24 × 10^−6^	2.9663	2.2125
Urinary	LM.Cannabis_x_Herb.THC_x_Daily.Interpol.	35.4475	1.3384	0.5944	0.5074	2.2517	0.0263	21.5361	2.0829
Urinary	Cocaine	35.4475	0.2657	0.0590	0.4738	4.5041	1.56 × 10^−5^	2.7191	2.0031
Urinary	LM.Cannabis_x_Resin.THC_x_Daily.Interpol.	35.4475	0.6738	0.2664	0.4985	2.5291	0.0130	6.2987	1.9731
Multicystic Renal Dys	Cocaine	3.4655	0.4660	0.1063	0.8533	4.3857	2.50 × 10^−5^	2.6723	1.9630
Hydronephrosis	LM.Cannabis_x_Resin.THC_x_Daily.Interpol.	13.8455	1.1656	0.4627	0.8659	2.5189	0.0134	6.2645	1.9582
Hypospadias	Cocaine	19.2037	0.2230	0.0608	0.4534	3.6679	3.86 × 10^−4^	2.5043	1.7674
Posterior Ureth Valv	LMCannabis_Resin	1.3062	1.2729	0.5745	0.6880	2.2154	0.0289	10.2449	1.7331
Hydronephrosis	LMCannabis_Resin	13.8455	1.5445	0.7215	0.8639	2.1407	0.0346	9.6488	1.5671
Bladder Extr/Epispad	LMCannabis_Resin	0.6657	0.8171	0.3848	0.4608	2.1235	0.0361	9.5157	1.5282
Hydronephrosis	Resin	13.8455	1.9876	0.9624	0.8652	2.0652	0.0414	15.6631	1.4781
Urinary	Amphetamine	35.4475	0.1765	0.0622	0.4959	2.8377	0.0053	2.1095	1.4480
Bladder Extr/Epispad	Cocaine	0.6657	0.1511	0.0548	0.4405	2.7545	0.0068	2.0738	1.4166
Hydronephrosis	Amphetamine	13.8455	0.2789	0.1039	0.8283	2.6856	0.0083	2.0566	1.3940
Hypospadias	Amphetamine	19.2037	0.1577	0.0603	0.4667	2.6142	0.0103	2.0598	1.3754
Multicystic Renal Dys	Resin	3.4655	2.1084	1.0383	0.9334	2.0306	0.0448	15.1060	1.3692
Bilat Renal Agenesis	Cocaine	1.3617	0.2017	0.0793	0.6368	2.5443	0.0122	2.0018	1.3404
Posterior Ureth Valv	Annual_Alcohol	1.3062	0.0958	0.0325	0.6537	2.9450	0.0039	1.5462	1.2646
Multicystic Renal Dys	Amphetamine	3.4655	0.2537	0.1129	0.9005	2.2464	0.0265	1.9067	1.2203

**Table 2:** Geotemporospatial Regressions for Uronephrological Congenital Anomalies as a Group.

Parameter Values			Model Parameters		
Parameter	Estimate (C.I.)	*p*-Value	Parameter	Value	Significance
***Additive***					
*Rate ~ Tobacco + Alcohol + LM.Cannabis_x_Herb.THC_x_Daily.Interpol. + LM.Cannabis_x_Resin.THC_x_Daily.Interpol. + LM.Cannabis_x_Herb.THC + Daily.Interpol. + Amphetamines + Cocaine + Income*					
Tobacco	0.06 (0.04, 0.07)	8.09 × 10^−12^	rho	−0.5677	2.99 × 10^−6^
Alcohol	0.03 (0, 0.06)	0.0394	lambda	0.4206	1.45 × 10^−5^
LM.Cannabis_x_Herb.THC_x_Daily.Interpol.	−4.7 (−5.97, −3.43)	4.05 × 10^−13^			
LM.Cannabis_x_Herb.THC	14.1 (10.53, 17.67)	7.84 × 10^−15^			
Cocaine	0.23 (0.12, 0.33)	2.82 × 10^−5^			
Income	0 (0, 0)	1.23 × 10^−14^			
***Interactive***					
Rate ~ Tobacco * LM.Cannabis_x_Herb.THC_x_Daily.Interpol. * LM.Cannabis_x_Resin.THC_x_Daily.Interpol. + LM.Cannabis_x_Resin.THC + Alcohol + Daily.Interpol. + Amphetamines + Cocaine + Income					
Tobacco	−0.04 (−0.07, −0.01)	0.0067	phi	6.1554	0.0464
LM.Cannabis_x_Resin.THC_x_Daily.Interpol.	−11.75 (−15.66, −7.83)	4.18 × 10^−9^	rho	−0.5643	8.32 × 10^−7^
Daily.Interpol.	19.94 (2.57, 37.3)	0.024429	lambda	0.5731	6.79 × 10^−10^
Tobacco: LM.Cannabis_x_Resin.THC_x_Daily.Interpol.	0.51 (0.34, 0.67)	3.07 × 10^−9^			
LM.Cannabis_x_Herb.THC_x_Daily.Interpol.: LM.Cannabis_x_Resin.THC_x_Daily.Interpol.	53.87 (26.64, 81.11)	0.0001			
Tobacco: LM.Cannabis_x_Herb.THC_x_Daily.Interpol.: LM.Cannabis_x_Resin.THC_x_Daily.Interpol.	−2.33 (−3.51, −1.14)	0.0001			
***1 Lag***					
Rate ~ Tobacco * LM.Cannabis_x_Herb.THC_x_Daily.Interpol. + Daily.Interpol. + LM.Cannabis_x_Resin.THC_x_Daily.Interpol. + Alcohol + Daily.Interpol. + Amphetamines + Cocaine + Income					
Tobacco	0.04 (0.02, 0.07)	0.0003	rho	0.2013	0.497
Daily.Interpol.	−31.9 (−53.26, −10.54)	0.0033	lambda	0.0696	0.782
LM.Cannabis_x_Resin.THC_x_Daily.Interpol.	0.85 (0.18, 1.53)	0.0130			
Alcohol	0.07 (0.02, 0.12)	0.0106			
Cocaine	0.52 (0.28, 0.75)	1.52 × 10^−5^			
Income	0 (0, 0)	0.0001			

Legend: lm—linear modelling; Left Hand Side—Dependent Variable; Right Hand side—List of independent covariates; ~—Separator between left and right hand sides of a model formula; +—Additive relationship between covariates; *—Interactive relationship between covariates—includes additive relationships; AUD—Alcohol Use Disorder; phi—Random error coefficient; psi—Serial correlation coefficient.; rho—Spatial error coefficient; lambda—Spatial error autocorrelation coefficient.

**Table 3:** Geotemporospatial Regressions for Multicystic Renal Disease Congenital Anomalies.

Parameter Values			Model Parameters		
Parameter	Estimate (C.I.)	*p*-Value	Parameter	Value	Significance
***Additive***					
*Rate ~ Tobacco + Alcohol + LM.Cannabis_x_Herb.THC_x_Daily.Interpol. + LM.Cannabis_x_Resin.THC_x_Daily.Interpol. + LM.Cannabis_x_Resin.THC + Daily.Interpol. + Amphetamines + Cocaine + Income*					
Tobacco	0.12 (0.06, 0.18)	9.17 × 10^−5^	psi	0.5495	4.92 × 10^−10^
LM.Cannabis_x_Resin.THC	1.17 (0.11, 2.23)	0.0310	rho	−0.5777	0.000168
Income	0 (0, 0)	0.0001	lambda	0.5010	0.000692
***Interactive***					
Rate ~ Tobacco * LM.Cannabis_x_Resin.THC_x_Daily.Interpol. + Daily.Interpol. + LM.Cannabis_x_Herb.THC_x_Daily.Interpol. + LM.Cannabis_x_Resin.THC + Alcohol + Amphetamines + Cocaine + Income					
Tobacco	0.12 (0.06, 0.18)	9.17 × 10^−5^	psi	0.5495	4.92 × 10^−10^
LM.Cannabis_x_Resin.THC	1.17 (0.11, 2.23)	0.0310	rho	−0.5777	0.000168
Income	0 (0, 0)	0.0001	lambda	0.5010	0.000692
***2 Lags***					
Rate ~ Tobacco * LM.Cannabis_x_Resin.THC + Daily.Interpol. + LM.Cannabis_x_Resin.THC_x_Daily.Interpol. + LM.Cannabis_x_Herb.THC_x_Daily.Interpol. + Alcohol + + Amphetamines + Cocaine + Income					
Tobacco	0.06 (0.02, 0.1)	0.0060	psi	0.5834	1.62 × 10^−9^
LM.Cannabis_x_Resin.THC	3.9 (1.97, 5.84)	7.72 × 10^−5^	rho	0.7044	3.78 × 10^−16^
Daily.Interpol.	74.8 (28.64, 120.96)	0.0015	lambda	−0.6093	7.22 × 10^−08^
LM.Cannabis_x_Herb.THC_x_Daily.Interpol.	−7.05 (−12.74, −1.36)	0.0152			
Amphetamines	−0.27 (−0.53, 0)	0.0493			

Legend—See Table 2.

**Table 4:** Geotemporospatial Regressions for Bilateral Renal Agenesis Congenital Anomalies.

Parameter Values			Model Parameters		
Parameter	Estimate (C.I.)	*p*-Value	Parameter	Value	Significance
***Additive***					
*Rate ~ Tobacco + Alcohol + LM.Cannabis_x_Herb.THC_x_Daily.Interpol. + LM.Cannabis_x_Resin.THC_x_Daily.Interpol. + LM.Cannabis_x_Resin.THC + Resin + Daily.Interpol. + Herb + Amphetamines + Cocaine + Income*					
Alcohol	0.13 (0.07, 0.18)	3.62 × 10^−6^	rho	0.6238	6.72 × 10^−10^
Income	0 (0, 0)	1.10 × 10^−8^	lambda	−0.5490	1.92 × 10^−6^
***Interactive***					
Rate ~ Tobacco * LM.Cannabis_x_Resin.THC_x_Daily.Interpol. + Daily.Interpol. + LM.Cannabis_x_Herb.THC_x_Daily.Interpol. + LM.Cannabis_x_Resin.THC + Alcohol + Herb + Amphetamines + Cocaine + Income					
LM.Cannabis_x_Resin.THC_x_Daily.Interpol.	4.06 (0.57, 7.55)	0.0228	rho	0.6314	2.39 × 10^−11^
Alcohol	0.14 (0.09, 0.2)	4.66 × 10^−7^	lambda	−0.5346	1.34 × 10^−6^
Income	0 (0, 0)	1.49 × 10^−8^			
LM.Cannabis_x_Herb.THC: LM.Cannabis_x_Resin.THC_x_Daily.Interpol.	−0.15 (−0.3, −0.01)	0.0327			
***2 Lags***					
Rate ~ Tobacco * Daily.Interpol. + LM.Cannabis_x_Resin.THC_x_Daily.Interpol. + LM.Cannabis_x_Herb.THC_x_Daily.Interpol. + LM.Cannabis_x_Resin.THC + Alcohol + Amphetamines + Cocaine + Income					
LM.Cannabis_x_Resin.THC	2.33 (0.83, 3.83)	0.0023	rho	0.5579	6.16 × 10^−5^
Alcohol	0.18 (0.11, 0.24)	6.12 × 10^−8^	lambda	−0.4473	0.00217
Cocaine	0.58 (0.26, 0.9)	0.0004			
Resin	0 (0, 0)	0.0033			
Tobacco: Daily.Interpol.	−1.87 (−2.9, −0.84)	0.0004			

Legend—See Table 2.

**Table 5:** Geotemporospatial Regressions for Hydronephrosis Congenital Anomalies.

Parameter Values			Model Parameters		
Parameter	Estimate (C.I.)	*p*-Value	Parameter	Value	Significance
***Additive***					
*Rate ~ Tobacco + Alcohol + Daily.Interpol. + LM.Cannabis_x_Herb.THC_x_Daily.Interpol. + LM.Cannabis_x_Resin.THC_x_Daily.Interpol. + Herb + Amphetamines + Cocaine + Income*					
Tobacco	−0.04 (−0.07, 0)	0.0283	psi	0.4594	3.24 × 10^−7^
Daily.Interpol.	67.26 (23.46, 111.06)	0.0026	rho	0.4513	0.00694
LM.Cannabis_x_Herb.THC_x_Daily.Interpol.	−9.26 (−16.96, −1.56)	0.0185	lambda	−0.5073	0.00149
Herb	12.02 (6.1, 17.94)	6.95 × 10^−5^			
***Interactive***					
Rate ~ Tobacco * Daily.Interpol. + Resin + Herb + LM.Cannabis_x_Herb.THC_x_Daily.Interpol. + LM.Cannabis_x_Resin.THC_x_Daily.Interpol. + Alcohol + Amphetamines + Cocaine + Income					
Tobacco	−0.05 (−0.09, −0.01)	0.0064	psi	0.5095	1.42 × 10^−9^
Herb	9.93 (4.34, 15.53)	0.0005	rho	0.4324	0.0151
LM.Cannabis_x_Herb.THC_x_Daily.Interpol.	−5.24 (−9.59, −0.89)	0.0182	lambda	−0.4830	0.00603
Tobacco: Daily.Interpol.	2.9 (1.29, 4.51)	0.0004			
***1 Lag***					
Rate ~ Tobacco + Herb * Resin + LM.Cannabis_x_Resin.THC_x_Daily.Interpol. * Daily.Interpol. + Herb + LM.Cannabis_x_Herb.THC_x_Daily.Interpol. + Alcohol + Amphetamines + Cocaine + Income					
Herb	6.01 (2.26, 9.76)	0.0017	psi	0.5117	1.30E^−9^
Cocaine	0.35 (0.1, 0.59)	0.0058	rho	−0.5342	0.00382
			lambda	0.4013	0.0324
***2 Lags***					
Rate ~ Tobacco * Herb + LM.Cannabis_x_Resin.THC_x_Daily.Interpol. + Daily.Interpol. + Resin + Alcohol + Amphetamines + Cocaine + Income					
Resin	−2.46 (−4.02, −0.9)	0.0020	psi	0.8338	< 2.22 × 10^−16^
Tobacco: Herb	−0.25 (−0.42, −0.08)	0.0033	rho	0.6650	6.20 × 10^−13^
			lambda	−0.5945	5.88 × 10^−7^

Legend—See Table 2.

**Table 6:** Geotemporospatial Regressions for Congenital Posterior Urethral Valve Congenital Anomalies.

Parameter Values			Model Parameters		
Parameter	Estimate (C.I.)	*p*-Value	Parameter	Value	Significance
***Additive***					
*Rate ~ Tobacco + Alcohol + LM.Cannabis_x_Herb.THC_x_Daily.Interpol. + LM.Cannabis_x_Resin.THC_x_Daily.Interpol. + LM.Cannabis_x_Resin.THC + Daily.Interpol. + Resin + Herb + Amphetamines + Cocaine + Income*					
Tobacco	0.04 (0.02, 0.06)	0.0001	psi	0.2311	0.0177
Alcohol	0.15 (0.08, 0.22)	9.98 × 10^−6^	rho	−0.5195	6.58 × 10^−5^
LM.Cannabis_x_Herb.THC_x_Daily.Interpol.	−10.18 (−14.3, −6.06)	1.29 × 10^−6^	lambda	0.5252	2.31 × 10^−5^
LM.Cannabis_x_Resin.THC_x_Daily.Interpol.	3.09 (1.55, 4.64)	8.82 × 10^−5^			
Herb	4.77 (1.87, 7.67)	0.0013			
Amphetamines	−0.19 (−0.36, −0.03)	0.0218			
Cocaine	0.51 (0.3, 0.71)	1.54 × 10^−6^			
***Interactive***					
Rate ~ Tobacco * Daily.Interpol. + LM.Cannabis_x_Herb.THC_x_Daily.Interpol. + LM.Cannabis_x_Resin.THC_x_Daily.Interpol. + Herb + Alcohol + Amphetamines + Cocaine + Income					
Tobacco	0.04 (0.02, 0.06)	0.0001	psi	0.2311	0.0177
LM.Cannabis_x_Herb.THC_x_Daily.Interpol.	−10.18 (−14.3, −6.06)	1.29 × 10^−6^	rho	−0.5195	6.58 × 10^−5^
LM.Cannabis_x_Resin.THC_x_Daily.Interpol.	3.09 (1.55, 4.64)	8.82 × 10^−5^	lambda	0.5252	2.33 × 10^−5^
Herb	4.77 (1.87, 7.67)	0.0013			
Alcohol	0.15 (0.08, 0.22)	9.98 × 10^−6^			
Amphetamines	−0.19 (−0.36, −0.03)	0.0218			
Cocaine	0.51 (0.3, 0.71)	1.54 × 10^−6^			
***1 Lag***					
Rate ~ Tobacco * LM.Cannabis_x_Resin.THC_x_Daily.Interpol. + Daily.Interpol. + LM.Cannabis_x_Herb.THC_x_Daily.Interpol. + LM.Cannabis_x_Herb.THC + Alcohol + Daily.Interpol. + Amphetamines + Cocaine + Income					
Tobacco	0.07 (0.02, 0.11)	0.0027	psi	0.3419	0.0013
LM.Cannabis_x_Resin.THC_x_Daily.Interpol.	2.65 (0.59, 4.71)	0.0114	rho	−0.4716	0.00201
Daily.Interpol.	91.2 (37.3, 145.1)	0.0009	lambda	0.5018	0.000476
LM.Cannabis_x_Herb.THC_x_Daily.Interpol.	−18.6 (−28.09, −9.11)	0.0001			
LM.Cannabis_x_Herb.THC	23.1 (11.16, 35.04)	0.0002			
Cocaine	−0.57 (−0.97, −0.16)	0.0057			
Income	0 (0, 0)	0.0036			
***2 Lags***					
Rate ~ Tobacco * LM.Cannabis_x_Resin.THC_x_Daily.Interpol. + Daily.Interpol. + LM.Cannabis_x_Herb.THC_x_Daily.Interpol. + LM.Cannabis_x_Herb.THC + Alcohol + Daily.Interpol. + Amphetamines + Cocaine + Income					
Tobacco	0.06 (0.01, 0.1)	0.0241	psi	0.3206	0.00367
LM.Cannabis_x_Resin.THC_x_Daily.Interpol.	3.06 (0.45, 5.67)	0.0217	rho	−0.3717	0.06365
LM.Cannabis_x_Herb.THC_x_Daily.Interpol.	−7.99 (−13.97, −2.01)	0.0087	lambda	0.4126	0.0323

Legend—See Table 2.

**Table 7:** E-Values from Multivariable Panel Regression Models.

Anomaly	Term	*p*-Value	E-Value Estimate	Lower Bound E-Value
				
Urinary	***Additive***			
	LM.Cannabis_x_Herb.THC_x_Daily.Interpol.	0.0012	9.03	3.23
	LM.Cannabis_x_Herb.THC	1.91 × 10^−5^	6.69 × 10^8^	1.22 × 10^5^
	***Interactive***			
	LM.Cannabis_x_Herb.THC_x_Daily.Interpol.	0.0165	15.85	2.38
	LM.Cannabis_x_Resin.THC	0.0079	67.00	4.73
	***2 Lags***			
	Tobacco: Resin	6.32 × 10^−5^	2.86	2.02
Multicystic Renal Disease	***Additive***			
	LM.Cannabis_x_Resin.THC	2.61 × 10^−8^	21.28	9.43
	***Interactive***			
	Daily.Interpol.	0.0498	6.23 × 10^63^	13.74
	Tobacco: Daily.Interpol.: LM.Cannabis_x_Resin.THC_x_Daily.Interpol.	0.0063	1.61 × 10^5^	56.63
	***2 Lags***			
	Daily.Interpol.	0.0433	1.18 × 10^103^	1.15 × 10^5^
	LM.Cannabis_x_Resin.THC	0.0097	606.01	8.35
	LM.Cannabis_x_Herb.THC_x_Daily.Interpol.	0.0269	59.95	2.51
Bilateral Renal Agenesis	***Additive***			
	Resin	0.0273	6.58	1.62
	Daily.Interpol.	0.0003	1.21 × 10^17^	1.95 × 10^8^
	***Interactive***			
	Daily.Interpol.	5.10 × 10^−8^	3.41 × 10^122^	3.30 × 10^81^
	***2 Lags***			
	LM.Cannabis_x_Resin.THC	4.60 × 10^−15^	42.81	22.36
Hydronephrosis	***Additive***			
	Daily.Interpol.	5.45 × 10^−6^	5.81 × 10^28^	1.24 × 10^17^
	LM.Cannabis_x_Herb.THC_x_Daily.Interpol.	0.0004	7.24	3.13
	Herb	0.0002	6.62 × 10^4^	303.56
	***Interactive***			
	Daily.Interpol.	0.0038	1.45 × 10^91^	1.36 × 10^31^
	Resin	0.0046	2.73 × 10^2^	9.28
	Herb	1.75 × 10^−7^	3.51 × 10^9^	2.01 × 10^6^
	LM.Cannabis_x_Herb.THC_x_Daily.Interpol.	0.0040	11.7	3.06
	***2 Lags***			
	Herb	1.87 × 10^−5^	2.33 × 10^7^	2.03 × 10^4^
Posterior Urethral Valve	***Additive***			
	LM.Cannabis_x_Resin.THC_x_Daily.Interpol.	2.25 × 10^−7^	10.52	5.47
	***Interactive***			
	LM.Cannabis_x_Resin.THC_x_Daily.Interpol.	3.38 × 10^−6^	2.65	2.02
	LM.Cannabis_x_Resin.THC	2.66 × 10^−10^	43.56	17.85
	***2 Lags***			
	Daily.Interpol.: LM.Cannabis_x_Resin.THC_x_Daily.Interpol.	0.0481	8.15 × 10^4^	1.89

**Table 8:** E-Values from Spatial Models.

Anomaly	Term	*p*-Value	E-Value Estimate	Lower Bound E-Value
Urinary	***Additive***			
	LM.Cannabis_x_Herb.THC	7.84 × 10^−15^	2.18 × 10^13^	1.14 × 10^10^
	***Interactive***			
	Daily.Interpol.	0.0244	5.96 × 10^17^	285.07
	Tobacco: LM.Cannabis_x_Resin.THC_x_Daily.Interpol.	3.07 × 10^−9^	5.01	3.38
	LM.Cannabis_x_Herb.THC_x_Daily.Interpol.: LM.Cannabis_x_Resin.THC_x_Daily.Interpol.	0.0001	3.34 × 10^47^	4.98 × 10^23^
	***1 Lag***			
	LM.Cannabis_x_Resin.THC_x_Daily.Interpol.	0.0130	8.58	2.10
Multicystic Renal Disease	***Additive***			
	LM.Cannabis_x_Resin.THC	0.0310	9.68	1.60
	***Interactive***			
	LM.Cannabis_x_Resin.THC	0.0310	9.68	1.60
	***2 Lags***			
	LM.Cannabis_x_Resin.THC	7.72 × 10^−5^	6.62 × 10^3^	119.49
	Daily.Interpol.	0.0015	6.01 × 10^67^	1.67 × 10^26^
Bilateral Renal Agenesis	***Interactive***			
	LM.Cannabis_x_Resin.THC_x_Daily.Interpol.	0.0228	3.54 × 10^3^	5.18
	***2 Lags***			
	LM.Cannabis_x_Resin.THC	0.0023	162.57	9.09
Hydronephrosis	***Additive***			
	Daily.Interpol.	0.0026	82.56	7.02
	Herb	6.95 × 10^−5^	4.51 × 10^8^	3.52 × 10^4^
	***Interactive***			
	Herb	0.0005	6.89 × 10^6^	1.46 × 10^3^
	Tobacco: Daily.Interpol.	0.0004	161.31	13.62
	***1 Lag***			
	Herb	0.0017	1.23 × 10^5^	1258.11
Posterior Urethral Valve	***Additive***			
	LM.Cannabis_x_Resin.THC_x_Daily.Interpol.	8.82 × 10^−5^	954.95	43.51
	Herb	0.0013	2.74 × 10^5^	83.66
	***Interactive***			
	LM.Cannabis_x_Resin.THC_x_Daily.Interpol.	8.82 × 10^−5^	955.32	43.52
	Herb	0.0013	2.73 × 10^4^	83.68
	***1 Lag***			
	LM.Cannabis_x_Resin.THC_x_Daily.Interpol.	0.0114	252.91	5.45
	Daily.Interpol.	0.0009	5.23 × 10^72^	1.08 × 10^30^
	LM.Cannabis_x_Herb.THC	0.0002	4.09 × 10^18^	1.39 × 10^9^
	***2 Lags***			
	LM.Cannabis_x_Resin.THC_x_Daily.Interpol.	0.0217	365.38	3.75

**Table 9:** All E-Values Ordered by Minimum E-Value.

No.	Anomaly	Regression	Model Type	Term	*p*-Value	E-Value Estimate	Lower Bound E-Value
1	Bilateral Renal Agenesis	Panel	Interactive	Daily.Interpol.	5.10 × 10^−8^	3.41 × 10^122^	3.30 × 10^81^
2	Hydronephrosis	Panel	Interactive	Daily.Interpol.	0.0038	1.45 × 10^91^	1.36 × 10^31^
3	Posterior Urethral Valve	Spatial	1 Lag	Daily.Interpol.	0.0009	5.23 × 10^72^	1.08 × 10^30^
4	Multicystic Renal Disease	Spatial	2 Lags	Daily.Interpol.	0.0015	6.01 × 10^67^	1.67 × 10^26^
5	Urinary	Spatial	Interactive	LM.Cannabis_x_Herb.THC_x_Daily.Interpol.: LM.Cannabis_x_Resin.THC_x_Daily.Interpol.	0.0001	3.34 × 10^47^	4.98 × 10^23^
6	Hydronephrosis	Panel	Additive	Daily.Interpol.	5.45 × 10^−6^	5.81 × 10^28^	1.24 × 10^17^
7	Urinary	Spatial	Additive	LM.Cannabis_x_Herb.THC	7.84 × 10^−15^	2.18 × 10^13^	1.14 × 10^10^
8	Posterior Urethral Valve	Spatial	1 Lag	LM.Cannabis_x_Herb.THC	0.0002	4.09 × 10^18^	1.39 × 10^9^
9	Bilateral Renal Agenesis	Panel	Additive	Daily.Interpol.	0.0003	1.21 × 10^17^	1.95 × 10^8^
10	Hydronephrosis	Panel	Interactive	Herb	1.75 × 10^−7^	3.51 × 10^9^	2.01 × 10^6^
11	Urinary	Panel	Additive	LM.Cannabis_x_Herb.THC	1.91 × 10^−5^	6.69 × 10^8^	1.22 × 10^5^
12	Multicystic Renal Disease	Panel	2 Lags	Daily.Interpol.	0.0433	1.18 × 10^103^	1.15 × 10^5^
13	Hydronephrosis	Spatial	Additive	Herb	6.95 × 10^−5^	4.51 × 10^8^	3.52 × 10^4^
14	Hydronephrosis	Panel	2 Lags	Herb	1.87 × 10^−5^	2.33 × 10^7^	2.03 × 10^4^
15	Hydronephrosis	Spatial	Interactive	Herb	0.0005	6.89 × 10^6^	1.46 × 10^3^
16	Hydronephrosis	Spatial	1 Lag	Herb	0.0017	1.23 × 10^5^	1258.11
17	Hydronephrosis	Panel	Additive	Herb	0.0002	6.62 × 10^4^	303.56
18	Urinary	Spatial	Interactive	Daily.Interpol.	0.0244	5.96 × 10^17^	285.07
19	Multicystic Renal Disease	Spatial	2 Lags	LM.Cannabis_x_Resin.THC	7.72 × 10^−5^	6.62 × 10^3^	119.49
20	Posterior Urethral Valve	Spatial	Interactive	Herb	0.0013	2.73 × 10^4^	83.68
21	Posterior Urethral Valve	Spatial	Additive	Herb	0.0013	2.74 × 10^5^	83.66
22	Multicystic Renal Disease	Panel	Interactive	Tobacco: Daily.Interpol.: LM.Cannabis_x_Resin.THC_x_Daily.Interpol.	0.0063	1.61 × 10^5^	56.63
23	Posterior Urethral Valve	Spatial	Interactive	LM.Cannabis_x_Resin.THC_x_Daily.Interpol.	8.82 × 10^-5^	955.32	43.52
24	Posterior Urethral Valve	Spatial	Additive	LM.Cannabis_x_Resin.THC_x_Daily.Interpol.	8.82 × 10^−5^	954.95	43.51
25	Bilateral Renal Agenesis	Panel	2 Lags	LM.Cannabis_x_Resin.THC	4.60 × 10^−15^	42.81	22.36
26	Posterior Urethral Valve	Panel	Interactive	LM.Cannabis_x_Resin.THC	2.66 × 10^−10^	43.56	17.85
27	Multicystic Renal Disease	Panel	Interactive	Daily.Interpol.	0.0498	6.23 × 10^63^	13.74
28	Hydronephrosis	Spatial	Interactive	Tobacco: Daily.Interpol.	0.0004	161.31	13.62
29	Multicystic Renal Disease	Panel	Additive	LM.Cannabis_x_Resin.THC	2.61 × 10^−8^	21.28	9.43
30	Hydronephrosis	Panel	Interactive	Resin	0.0046	273.00	9.28
31	Bilateral Renal Agenesis	Spatial	2 Lags	LM.Cannabis_x_Resin.THC	0.0023	162.57	9.09
32	Multicystic Renal Disease	Panel	2 Lags	LM.Cannabis_x_Resin.THC	0.0097	606.01	8.35
33	Hydronephrosis	Spatial	Additive	Daily.Interpol.	0.0026	82.56	7.02
34	Posterior Urethral Valve	Panel	Additive	LM.Cannabis_x_Resin.THC_x_Daily.Interpol.	2.25 × 10^−7^	10.52	5.47
35	Posterior Urethral Valve	Spatial	1 Lag	LM.Cannabis_x_Resin.THC_x_Daily.Interpol.	0.0114	252.91	5.45
36	Bilateral Renal Agenesis	Spatial	Interactive	LM.Cannabis_x_Resin.THC_x_Daily.Interpol.	0.0228	3540.00	5.18
37	Urinary	Panel	Interactive	LM.Cannabis_x_Resin.THC	0.0079	67.00	4.73
38	Posterior Urethral Valve	Spatial	2 Lags	LM.Cannabis_x_Resin.THC_x_Daily.Interpol.	0.0217	365.38	3.75
39	Urinary	Spatial	Interactive	Tobacco: LM.Cannabis_x_Resin.THC_x_Daily.Interpol.	3.07 × 10^−9^	5.01	3.38
40	Urinary	Panel	Additive	LM.Cannabis_x_Herb.THC_x_Daily.Interpol.	0.0012	9.03	3.23
41	Hydronephrosis	Panel	Additive	LM.Cannabis_x_Herb.THC_x_Daily.Interpol.	0.0004	7.24	3.13
42	Hydronephrosis	Panel	Interactive	LM.Cannabis_x_Herb.THC_x_Daily.Interpol.	0.0040	11.7	3.06
43	Multicystic Renal Disease	Panel	2 Lags	LM.Cannabis_x_Herb.THC_x_Daily.Interpol.	0.0269	59.95	2.51
44	Urinary	Panel	Interactive	LM.Cannabis_x_Herb.THC_x_Daily.Interpol.	0.0165	15.85	2.38
45	Urinary	Spatial	1 Lag	LM.Cannabis_x_Resin.THC_x_Daily.Interpol.	0.0130	8.58	2.10
46	Posterior Urethral Valve	Panel	Interactive	LM.Cannabis_x_Resin.THC_x_Daily.Interpol.	3.38 × 10^−6^	2.65	2.02
47	Urinary	Panel	2 Lags	Tobacco: Resin	6.32 × 10^−5^	2.86	2.02
48	Posterior Urethral Valve	Panel	2 Lags	Daily.Interpol.: LM.Cannabis_x_Resin.THC_x_Daily.Interpol.	0.0481	81500.00	1.89
49	Bilateral Renal Agenesis	Panel	Additive	Resin	0.0273	6.58	1.62
50	Multicystic Renal Disease	Spatial	Additive	LM.Cannabis_x_Resin.THC	0.0310	9.68	1.60
51	Multicystic Renal Disease	Spatial	Interactive	LM.Cannabis_x_Resin.THC	0.0310	9.68	1.60

The E-values may be extracted alone and listed and ordered as shown in Table 10. Here, it is noted that 45/51 (88.2%) E-value estimates exceed 9 and are thus in the high range [110], and all 51/51 (100%) exceed the threshold of causality at 1.25 [109]. Moreover, 31/51 (60.8%) mEV’s exceed nine and therefore fall into the high range and 51/51 (100%) exceed the threshold for causality.

Table 9 may also be ordered by anomaly as shown in Table 11 and summarized in Table 12. Table 12 is also ordered by descending median minimum E-value. The table is headed up by hydronephrosis and posterior urethral valve. It is noted that the median mEV’s are relatively high for the five anomalies studied. 

Table 9 may also be ordered by the regression term and grouped according to the major covariate between daily cannabis use, and the THC concentration of cannabis herb and resin. This is defined by the extra column which has now been added headed “Group” in Table 13. This table may then be summarized by the primary cannabis metric as shown in Table 14. 

These results may then be compared using the Wilcoxson test a indicated in Table 15. One notes immediately that most of the apparent between group differences noted in Table 14 are statistically highly significantly different.

**Table 11:** E-Values by Anomaly.

No.	Anomaly	Regression	Model Type	Term	*p*-Value	E-Value Estimate	Lower Bound E-Value
1	Bilateral Renal Agenesis	Panel	Interactive	Daily.Interpol.	5.10 × 10^−8^	3.41 × 10^122^	3.30 × 10^81^
2	Bilateral Renal Agenesis	Panel	Additive	Daily.Interpol.	0.0003	1.21 × 10^17^	1.95 × 10^8^
3	Bilateral Renal Agenesis	Panel	2 Lags	LM.Cannabis_x_Resin.THC	4.60 × 10^−15^	42.81	22.36
4	Bilateral Renal Agenesis	Spatial	2 Lags	LM.Cannabis_x_Resin.THC	0.0023	162.57	9.09
5	Bilateral Renal Agenesis	Spatial	Interactive	LM.Cannabis_x_Resin.THC_x_Daily.Interpol.	0.0228	3540.00	5.18
6	Bilateral Renal Agenesis	Panel	Additive	Resin	0.0273	6.58	1.62
7	Hydronephrosis	Panel	Interactive	Daily.Interpol.	0.0038	1.45 × 10^91^	1.36 × 10^31^
8	Hydronephrosis	Panel	Additive	Daily.Interpol.	5.45 × 10^-6^	5.81 × 10^28^	1.24 × 10^17^
9	Hydronephrosis	Panel	Interactive	Herb	1.75 × 10^−7^	3.51 × 10^9^	2.01 × 10^6^
10	Hydronephrosis	Spatial	Additive	Herb	6.95 × 10^−5^	4.51 × 10^8^	3.52 × 10^4^
11	Hydronephrosis	Panel	2 Lags	Herb	1.87 × 10^−5^	2.33 × 10^7^	2.03 × 10^4^
12	Hydronephrosis	Spatial	Interactive	Herb	0.0005	6.89 × 10^6^	1.46 × 10^3^
13	Hydronephrosis	Spatial	1 Lag	Herb	0.0017	1.23 × 10^5^	1258.11
14	Hydronephrosis	Panel	Additive	Herb	0.0002	6.62 × 10^4^	303.56
15	Hydronephrosis	Spatial	Interactive	Tobacco: Daily.Interpol.	0.0004	161.31	13.62
16	Hydronephrosis	Panel	Interactive	Resin	0.0046	273.00	9.28
17	Hydronephrosis	Spatial	Additive	Daily.Interpol.	0.0026	82.56	7.02
18	Hydronephrosis	Panel	Additive	LM.Cannabis_x_Herb.THC_x_Daily.Interpol.	0.0004	7.24	3.13
19	Hydronephrosis	Panel	Interactive	LM.Cannabis_x_Herb.THC_x_Daily.Interpol.	0.0040	11.7	3.06
20	Multicystic Renal Disease	Spatial	2 Lags	Daily.Interpol.	0.0015	6.01 × 10^67^	1.67 × 10^26^
21	Multicystic Renal Disease	Panel	2 Lags	Daily.Interpol.	0.0433	1.18 × 10^103^	1.15 × 10^5^
22	Multicystic Renal Disease	Spatial	2 Lags	LM.Cannabis_x_Resin.THC	7.72 × 10^−5^	6.62 × 10^3^	119.49
23	Multicystic Renal Disease	Panel	Interactive	Tobacco: Daily.Interpol.: LM.Cannabis_x_Resin.THC_x_Daily.Interpol.	0.0063	1.61 × 10^5^	56.63
24	Multicystic Renal Disease	Panel	Interactive	Daily.Interpol.	0.0498	6.23 × 10^63^	13.74
25	Multicystic Renal Disease	Panel	Additive	LM.Cannabis_x_Resin.THC	2.61 × 10^−8^	21.28	9.43
26	Multicystic Renal Disease	Panel	2 Lags	LM.Cannabis_x_Resin.THC	0.0097	606.01	8.35
27	Multicystic Renal Disease	Panel	2 Lags	LM.Cannabis_x_Herb.THC_x_Daily.Interpol.	0.0269	59.95	2.51
28	Multicystic Renal Disease	Spatial	Additive	LM.Cannabis_x_Resin.THC	0.0310	9.68	1.60
29	Multicystic Renal Disease	Spatial	Interactive	LM.Cannabis_x_Resin.THC	0.0310	9.68	1.60
30	Posterior Urethral Valve	Spatial	1 Lag	Daily.Interpol.	0.0009	5.23 × 10^72^	1.08 × 10^30^
31	Posterior Urethral Valve	Spatial	1 Lag	LM.Cannabis_x_Herb.THC	0.0002	4.09 × 10^18^	1.39 × 10^9^
32	Posterior Urethral Valve	Spatial	Interactive	Herb	0.0013	2.73 × 10^4^	83.68
33	Posterior Urethral Valve	Spatial	Additive	Herb	0.0013	2.74 × 10^5^	83.66
34	Posterior Urethral Valve	Spatial	Interactive	LM.Cannabis_x_Resin.THC_x_Daily.Interpol.	8.82 × 10^−5^	955.32	43.52
35	Posterior Urethral Valve	Spatial	Additive	LM.Cannabis_x_Resin.THC_x_Daily.Interpol.	8.82 × 10^−5^	954.95	43.51
36	Posterior Urethral Valve	Panel	Interactive	LM.Cannabis_x_Resin.THC	2.66 × 10^−10^	43.56	17.85
37	Posterior Urethral Valve	Panel	Additive	LM.Cannabis_x_Resin.THC_x_Daily.Interpol.	2.25 × 10^−7^	10.52	5.47
38	Posterior Urethral Valve	Spatial	1 Lag	LM.Cannabis_x_Resin.THC_x_Daily.Interpol.	0.0114	252.91	5.45
39	Posterior Urethral Valve	Spatial	2 Lags	LM.Cannabis_x_Resin.THC_x_Daily.Interpol.	0.0217	365.38	3.75
40	Posterior Urethral Valve	Panel	Interactive	LM.Cannabis_x_Resin.THC_x_Daily.Interpol.	3.38 × 10^−6^	2.65	2.02
41	Posterior Urethral Valve	Panel	2 Lags	Daily.Interpol.: LM.Cannabis_x_Resin.THC_x_Daily.Interpol.	0.0481	81500.00	1.89
42	Urinary	Spatial	Interactive	LM.Cannabis_x_Herb.THC_x_Daily.Interpol.: LM.Cannabis_x_Resin.THC_x_Daily.Interpol.	0.0001	3.34 × 10^47^	4.98 × 10^23^
43	Urinary	Spatial	Additive	LM.Cannabis_x_Herb.THC	7.84 × 10^−15^	2.18 × 10^13^	1.14 × 10^10^
44	Urinary	Panel	Additive	LM.Cannabis_x_Herb.THC	1.91 × 10^−5^	6.69 × 10^8^	1.22 × 10^5^
45	Urinary	Spatial	Interactive	Daily.Interpol.	0.0244	5.96 × 10^17^	285.07
46	Urinary	Panel	Interactive	LM.Cannabis_x_Resin.THC	0.0079	67.00	4.73
47	Urinary	Spatial	Interactive	Tobacco: LM.Cannabis_x_Resin.THC_x_Daily.Interpol.	3.07 × 10^−9^	5.01	3.38
48	Urinary	Panel	Additive	LM.Cannabis_x_Herb.THC_x_Daily.Interpol.	0.0012	9.03	3.23
49	Urinary	Panel	Interactive	LM.Cannabis_x_Herb.THC_x_Daily.Interpol.	0.0165	15.85	2.38
50	Urinary	Spatial	1 Lag	LM.Cannabis_x_Resin.THC_x_Daily.Interpol.	0.0130	8.58	2.10
51	Urinary	Panel	2 Lags	Tobacco: Resin	6.32 × 10^−5^	2.86	2.02

**Table 12:** Summary of E-Values by Anomaly.

Anomaly	Number	Mean Minimum E-Value	Median Minimum E-Value	Min Minimum E-Value	Max Minimum E-Value	Mean E-Value Estimate	Median E-Value Estimate	Min E-Value Estimate	Max E-Value Estimate
Hydronephrosis	13	1.05 × 10^30^	1258.11	3.06	1.36 × 10^31^	1.12 × 10^90^	1.23 × 10^5^	7.24	1.45 × 10^91^
Posterior Urethral Valve	12	9.00 × 10^28^	30.68	1.89	1.08 × 10^30^	4.36 × 10^71^	955.14	2.65	5.23 × 10^72^
Bilateral Renal Agenesis	6	5.50 × 10^80^	15.73	1.62	3.30 × 10^81^	5.68 × 10^121^	1.85 × 10^3^	6.58	3.41 × 10^122^
Multicystic Renal Disease	10	1.67 × 10^25^	11.59	1.6	1.67 × 10^26^	1.18 × 10^102^	3.61 × 10^3^	9.68	1.18 × 10^103^
Urinary	10	4.98 × 10^22^	4.055	2.02	4.98 × 10^23^	3.34 × 10^46^	41.43	2.86	3.34 × 10^47^

**Table 13:** E-Values by Covariate.

No.	Anomaly	Regression	Model Type	Term	Group	*p*-Value	E-Value Estimate	Lower Bound E-Value
1	Bilateral Renal Agenesis	Panel	Interactive	Daily.Interpol.	Daily	5.10 × 10^−8^	3.41 × 10^122^	3.30 × 10^81^
2	Hydronephrosis	Panel	Interactive	Daily.Interpol.	Daily	0.0038	1.45 × 10^91^	1.36 × 10^31^
3	Posterior Urethral Valve	Spatial	1 Lag	Daily.Interpol.	Daily	0.0009	5.23 × 10^72^	1.08 × 10^30^
4	Multicystic Renal Disease	Spatial	2 Lags	Daily.Interpol.	Daily	0.0015	6.01 × 10^67^	1.67 × 10^26^
5	Hydronephrosis	Panel	Additive	Daily.Interpol.	Daily	5.45 × 10^−6^	5.81 × 10^28^	1.24 × 10^17^
6	Bilateral Renal Agenesis	Panel	Additive	Daily.Interpol.	Daily	0.0003	1.21 × 10^17^	1.95 × 10^8^
7	Multicystic Renal Disease	Panel	2 Lags	Daily.Interpol.	Daily	0.0433	1.18 × 10^103^	1.15 × 10^5^
8	Urinary	Spatial	Interactive	Daily.Interpol.	Daily	0.0244	5.96 × 10^17^	285.07
9	Multicystic Renal Disease	Panel	Interactive	Daily.Interpol.	Daily	0.0498	6.23 × 10^63^	13.74
10	Hydronephrosis	Spatial	Additive	Daily.Interpol.	Daily	0.0026	82.56	7.02
11	Posterior Urethral Valve	Panel	2 Lags	Daily.Interpol.: LM.Cannabis_x_Resin.THC_x_Daily.Interpol.	Daily	0.0481	81500.00	1.89
12	Hydronephrosis	Panel	Interactive	Herb	Herb	1.75 × 10^−7^	3.51 × 10^9^	2.01 × 10^6^
13	Hydronephrosis	Spatial	Additive	Herb	Herb	6.95 × 10^−5^	4.51 × 10^8^	3.52 × 10^4^
14	Hydronephrosis	Panel	2 Lags	Herb	Herb	1.87 × 10^−5^	2.33 × 10^7^	2.03 × 10^4^
15	Hydronephrosis	Spatial	Interactive	Herb	Herb	0.0005	6.89 × 10^6^	1.46 × 10^3^
16	Hydronephrosis	Spatial	1 Lag	Herb	Herb	0.0017	1.23 × 10^5^	1258.11
17	Hydronephrosis	Panel	Additive	Herb	Herb	0.0002	6.62 × 10^4^	303.56
18	Posterior Urethral Valve	Spatial	Interactive	Herb	Herb	0.0013	2.73 × 10^4^	83.68
19	Posterior Urethral Valve	Spatial	Additive	Herb	Herb	0.0013	2.74 × 10^5^	83.66
20	Urinary	Spatial	Additive	LM.Cannabis_x_Herb.THC	Herb	7.84 × 10^−15^	2.18 × 10^13^	1.14 × 10^10^
21	Posterior Urethral Valve	Spatial	1 Lag	LM.Cannabis_x_Herb.THC	Herb	0.0002	4.09 × 10^18^	1.39 × 10^9^
22	Urinary	Panel	Additive	LM.Cannabis_x_Herb.THC	Herb	1.91 × 10^−5^	6.69 × 10^8^	1.22 × 10^5^
23	Urinary	Panel	Additive	LM.Cannabis_x_Herb.THC_x_Daily.Interpol.	Herb	0.0012	9.03	3.23
24	Hydronephrosis	Panel	Additive	LM.Cannabis_x_Herb.THC_x_Daily.Interpol.	Herb	0.0004	7.24	3.13
25	Hydronephrosis	Panel	Interactive	LM.Cannabis_x_Herb.THC_x_Daily.Interpol.	Herb	0.0040	11.7	3.06
26	Multicystic Renal Disease	Panel	2 Lags	LM.Cannabis_x_Herb.THC_x_Daily.Interpol.	Herb	0.0269	59.95	2.51
27	Urinary	Panel	Interactive	LM.Cannabis_x_Herb.THC_x_Daily.Interpol.	Herb	0.0165	15.85	2.38
28	Urinary	Spatial	Interactive	LM.Cannabis_x_Herb.THC_x_Daily.Interpol.: LM.Cannabis_x_Resin.THC_x_Daily.Interpol.	Herb	0.0001	3.34 × 10^47^	4.98 × 10^23^
29	Multicystic Renal Disease	Spatial	2 Lags	LM.Cannabis_x_Resin.THC	Resin	7.72 × 10^−5^	6.62 × 10^3^	119.49
30	Bilateral Renal Agenesis	Panel	2 Lags	LM.Cannabis_x_Resin.THC	Resin	4.60 × 10^−15^	42.81	22.36
31	Posterior Urethral Valve	Panel	Interactive	LM.Cannabis_x_Resin.THC	Resin	2.66 × 10^−10^	43.56	17.85
32	Multicystic Renal Disease	Panel	Additive	LM.Cannabis_x_Resin.THC	Resin	2.61 × 10^−8^	21.28	9.43
33	Bilateral Renal Agenesis	Spatial	2 Lags	LM.Cannabis_x_Resin.THC	Resin	0.0023	162.57	9.09
34	Multicystic Renal Disease	Panel	2 Lags	LM.Cannabis_x_Resin.THC	Resin	0.0097	606.01	8.35
35	Urinary	Panel	Interactive	LM.Cannabis_x_Resin.THC	Resin	0.0079	67.00	4.73
36	Multicystic Renal Disease	Spatial	Additive	LM.Cannabis_x_Resin.THC	Resin	0.0310	9.68	1.60
37	Multicystic Renal Disease	Spatial	Interactive	LM.Cannabis_x_Resin.THC	Resin	0.0310	9.68	1.60
38	Posterior Urethral Valve	Spatial	Interactive	LM.Cannabis_x_Resin.THC_x_Daily.Interpol.	Resin	8.82 × 10^−5^	955.32	43.52
39	Posterior Urethral Valve	Spatial	Additive	LM.Cannabis_x_Resin.THC_x_Daily.Interpol.	Resin	8.82 × 10^−5^	954.95	43.51
40	Posterior Urethral Valve	Panel	Additive	LM.Cannabis_x_Resin.THC_x_Daily.Interpol.	Resin	2.25 × 10^−7^	10.52	5.47
41	Posterior Urethral Valve	Spatial	1 Lag	LM.Cannabis_x_Resin.THC_x_Daily.Interpol.	Resin	0.0114	252.91	5.45
42	Bilateral Renal Agenesis	Spatial	Interactive	LM.Cannabis_x_Resin.THC_x_Daily.Interpol.	Resin	0.0228	3540.00	5.18
43	Posterior Urethral Valve	Spatial	2 Lags	LM.Cannabis_x_Resin.THC_x_Daily.Interpol.	Resin	0.0217	365.38	3.75
44	Urinary	Spatial	1 Lag	LM.Cannabis_x_Resin.THC_x_Daily.Interpol.	Resin	0.0130	8.58	2.10
45	Posterior Urethral Valve	Panel	Interactive	LM.Cannabis_x_Resin.THC_x_Daily.Interpol.	Resin	3.38 × 10^−6^	2.65	2.02
46	Hydronephrosis	Panel	Interactive	Resin	Resin	0.0046	273.00	9.28
47	Bilateral Renal Agenesis	Panel	Additive	Resin	Resin	0.0273	6.58	1.62
48	Hydronephrosis	Spatial	Interactive	Tobacco: Daily.Interpol.	Daily	0.0004	161.31	13.62
49	Multicystic Renal Disease	Panel	Interactive	Tobacco: Daily.Interpol.: LM.Cannabis_x_Resin.THC_x_Daily.Interpol.	Resin	0.0063	1.61 × 10^+5^	56.63
50	Urinary	Spatial	Interactive	Tobacco: LM.Cannabis_x_Resin.THC_x_Daily.Interpol.	Resin	3.07 × 10^−9^	5.01	3.38
51	Urinary	Panel	2 Lags	Tobacco: Resin	Resin	6.32 × 10^−5^	2.86	2.02

**Table 14:** Summary of E-Values by Major Covariate.

Group	Number	Mean Minimum E-Value	Median Minimum E-Value	Minimum Minimum E-Value	Maximum Minimum E-Value	Mean E-Value Estimate	Median E-Value Estimate	Minimum E-Value Estimate	Maximum E-Value Estimate
Daily	12	2.75 × 10^80^	9.76 × 10^7^	1.89	3.30 × 10^81^	2.84 × 10^121^	3.12 × 10^63^	82.56	3.41 × 10^122^
Herb	17	2.93 × 10^22^	1258.11	2.38	4.98 × 10^23^	1.96 × 10^46^	2.74 × 10^5^	7.24	3.34 × 10^47^
Resin	22	17.20	5.46	1.6	119.49	7952.74	55.28	2.65	1.61 × 10^5^

**Table 15:** Wilcoxson Tests of Major Group Contrasts.

Comparison	W-Statistic	Alternative	*p*-Value
mEV, Daily_v_Herb	132	two.sided	0.1947
mEV, Daily_v_Resin	223	two.sided	0.0011
mEV, Herb_v_Resin	292	two.sided	0.0031
EVEstimate, Daily_v_Herb	165	two.sided	0.0043
EVEstimate, Daily_v_Resin	243	two.sided	6.82 × 10^−5^
EVEstimate, Herb_v_Resin	297	two.sided	0.0019

Legend: mEV—minimum E-Value; EVEstimate—E-value estimate.

## 4. Discussion

### 4.1. Main Results

The main results of this study is that all the UCAR’s identified are closely related to various metrics of cannabis exposure. Strong relationships were demonstrated on bivariate analysis which persisted after multivariable adjustment in inverse probability weighted panel models and thereby fulfilled the formal criteria of quantitative causal inference. These results were also confirmed for all five UCA’s studies by multivariable geospatial regression.

### 4.2. Detailed Main Results

A mapping analysis demonstrated that UCAR’s increased across Spain, Netherlands, Poland and France. Congenital posterior urethral valve rates increased in Spain, France, Belgium, Poland and Netherlands. Multicystic renal disease increased in Spain, France, Netherlands and Norway. A bivariate map study showed that UCAR’s and last month cannabis use x cannabis resin THC concentration increased simultaneously in France, Spain, Netherlands and Bulgaria. Bilateral renal agenesis increased along with last month cannabis use x cannabis resin THC concentration in Netherlands. Congenital posterior urethral valve increased along with last month cannabis use x cannabis resin THC concentration in Netherlands and France. Multicystic renal disease increased along with last month cannabis use x cannabis resin THC concentration in Netherlands and France. That is UCAR’s increased in countries with increased daily cannabis exposure.

When nations with increasing daily cannabis use were compared with those without the former group has a higher rate of UCA’s overall (*p* = 0.0073). When this comparison was made by UCA this relationship intensified (*p* = 8.73 × 10^−5^).

At bivariate analysis tobacco exposure was not related to UCA’s. Alcohol exposure was related to multicystic renal disease and posterior urethral valve. Cocaine and amphetamine were strongly related to most UCA’s. All UCA’s were strongly related to cannabis herb and resin THC concentrations (Figure 1 and Figure 2). All anomalies could be related to cannabis metrics on bivariate analysis with strength of association judged by the median minimum E-value (mEV) as hypospadias (174.71) > multicystic renal disease (5.37) > bilateral renal agenesis (5.05) > UCA’s (2.68) > hydronephrosis (2.21) > posterior urethral valve (1.73) > bladder exstrophy/epispadias (1.53) (Table 1).

At inverse probability weighted multivariable analysis terms including cannabis were significant for the following series of anomalies: UCA’s, multicystic renal disease, bilateral renal agenesis, hydronephrosis, congenital posterior urethral valves from P = 1.91 × 10^−5^, 2.61 × 10^−8^, 4.60 × 10^−15^, 4.60 × 10^−15^ and 2.66 × 10^−10^ (Supplementary Tables S8–S12). At geospatial analysis the same series of UCA’s were significantly related to cannabis from *p* = 7.84 × 10^−15^, 7.72 × 10^−5^, 0.0023, 6.95 × 10^−5^, and 8.82 × 10^−5^ (Table 2, Table 3, Table 4, Table 5 and Table 6). 

45/51 (88.2%) of E-value estimates and 31/51 (60.8%) of mEV’s exceeded nine and were thus in the high range [110]. 100% of both E-value estimates and mEV’s exceeded the threshold of causality at 1.25 (Table 10) [109]. At multivariable analysis the rank of strength of association was hydronephrosis > posterior urethral valve > bilateral renal agenesis > multicystic renal disease > UCA’s (Table 12). The rank of primary cannabis covariates was daily use > cannabis herb THC concentration > cannabis resin THC concentration (Table 14).

### 4.3. Choice of Anomalies

The selection of UCA’s chosen for detailed analysis was based upon their implication in prior reports as mentioned in the Introduction, the frequency of the anomaly and the clinical significance. Hypospadias and epispadias were not chosen as they are relatively minor anomalies. Bladder exstrophy was not studied further as it is very rare. 

### 4.4. Qualitative Causal Inference

It is worth considering how the present results perform against the Hill criteria of causality which are accepted criteria for assessing causal relationships [112]. These criteria were strength of association, consistency amongst studies, specificity, temporality, coherence with known data, biological plausibility, overresponse relationship, analogy with similar situations elsewhere and experimental confirmation. It is noted that the present study fulfills all of these criteria.

### 4.5. Quantitative Causal Inference

One of the major issues confronting the interpretation of observational studies is the issue of non-comparability of experimental groups. This issue can be addressed by inverse probability weighting which is the technique of choice in causal inference and has been widely adopted here in all panel models. This technique has the effect of transforming the analysis from that of an observational study only into a pseudorandomized quasi-experimental system from which it is entirely proper to draw causal inferences.

The other issue with which observational studies are frequently confronted is the potential for some uncontrolled extraneous confounding covariate to explain away the reported results. This issue is addressed by calculating the E-value which quantifies the degree of association required of such an hypothetical covariate with both the exposure of concern and the outcome of interest in order to obviate the reported apparently association. E-Values (expected values) in excess of nine are reported as being in the high range [110] and a cut-ff of 1.25 is usually applied as a threshold of a potentially causal effect [109]. Hence, the very elevated E-values reported in the present study are well above this range and qualify findings to be designated as causal in nature. 

Together with highly concordant results reported from several other jurisdictions and the strong theoretical mechanistic basis of these findings the highly significant results reported herein exclude the possibility that the results reported may be due to either chance (*p*-values) or bias (E-values).

### 4.6. Mechanisms

As noted in the Introduction cannabinoid genotoxicity is mediated by multiple pathways. These have been reviewed in detail elsewhere [1,2,3,4,5,6,7,8,9,14,15,23,24,25,43,54,55,113,114,115]. For our present purposes however it suffices to consider two main mechanistic classes, inhibition of the major morphogen gradients governing embryological morphogenesis and epigenomic mechanisms.

### 4.7. Cannabinoid Inhibition of Embryonic Morphogens

Many of the key morphogen gradients governing human embryogenesis are known to be disrupted by cannabinoids including sonic hedgehog [116], fibroblast growth factor [117,118], bone morphogenetic proteins [119,120,121] and retinoic acid [122,123,124].

In part this effects happens directly but it is also partly epigenomically mediated. Hence, for example genes such as GLI3, (Gli family zinc finger 3), MEGF8 (multiple EGF-like domains 8), TMEM107 (transmembrane protein 107) and BMP4 (bone morphogenetic protein 4) which are frequently cited in the epigenome-wide screen as being altered in cannabis dependence and withdrawal, are known to function in or around or antagonize sonic hedgehog signalling which is a critically important controller of embryonic morphogenesis across numerous tissues and organs.

### 4.8. Epigenomic Control of Uronephrological Gene Expression

Review of the supplementary material supplied in the recent epigenome-wide DNA methylation screen of Schrott and colleagues in both cannabis dependence and withdrawal [55] shows identified functional annotations on an Ingenuity Pathway Screen for renal agenesis (5 genes, page 311 in cannabis dependence), morphology of the renal tubule (8 genes, page 313), urinary tract cancer (84 genes, page 320), renal morphology (16 genes, page 322, *p* = 0.00216; 17 genes, *p* = 0.0022, page 322), formation of renal lesions (48 genes, page 322), abnormalities of the renal tubule (7 genes, page 323), congenital anomalies of the kidneys and urinary tract (9 genes, page 351 in cannabis withdrawal) and familial congenital anomalies of the kidney and urinary tract (8 genes, page 352; see Table 16).

Genes involved in male genital neoplasia were also notable increased (153 genes, page 279, *p* = 1.14 × 10^−8^; 151 genes, *p* = 1.60 × 10^−9^, page 279) and (60 genes, page 341 in cannabis withdrawal).

Functional annotations applicable to prostatic neoplasia were also identified (148 genes, page 279, *p* = 2.88 × 10^−8^ in cannabis dependence; 60 genes, page 341, *p* = 0.000675 in cannabis withdrawal; 59 genes, page 340, *p* = 0.000533), and adhesion of prostate cancer cells (1 gene, page 357, *p* = 0.00701, EPHA2). 

This link between cannabidiol and prostate cancer has been confirmed in recent epidemiological reports [1,2,3].

Female genital neoplasia was also linked by functional annotations including female genital adenocarcinoma (197 genes, page 272, *p* = 1.84 × 10^−11^ in cannabis dependence; 2.05 × 10^−11^, 215 genes, page 273; 216 genes, page 276, *p* = 8.95 × 10^−11^) and female genital tract cancer (78 genes, page 349 in cannabis withdrawal, *p* = 0.00303) and female genital neoplasm (79 genes, page 350). Breast and ovarian cancer were identified together by related sets of genes (133 genes, page 315, *p* = 0.000965 in cannabis dependence; 121 genes page 121, *p* = 0.000161).

This link between cannabidiol and ovarian cancer was confirmed in a recent epidemiological study [1,2,3].

### 4.9. Nephrogenic Genes in Renal Organoids

A recent detailed study of renal morphogenesis has been most revealing in terms of disclosing, and confirming genes critically involved in renal morphogenesis [125]. Inhibition of ROCK (Rho associated coiled-coil containing protein kinase) proved to be a key step in dramatically increasing the yield of embryonic stem cell differentiation into nephroblastic progenitor cells. Many genes involved in the induction of ciliopathies were also implicated in grossly abnormal nephrogenesis. Furthermore, the key notch ligand Jag1 (Jagged canonical notch ligand) was shown to potently inhibit differentiation into renal tubular cells.

Table 17 lists several of the genes from the notch pathway which were identified in the epigenomic EWAS of Schrott and colleagues [55]. RBPJ (Recombination Signal Binding Protein for Immunoglobulin Kappa J Region) and PSENEN (Presenilin enhancer, gamma secretase subunit, a key transmembrane effector of notch ligand cleavage and thus signal transduction) were prominently identified as shown.

Sonic hedgehog (shh) is another key morphogen involved in nephrogenesis [125,126]. Table 18 lists some of the EWAS hits from the Schrott study where shh pathway members were identified as being disrupted by cannabis exposure. Patched is the canonical shh receptor. SUFU (SUFU negative regulator of hedgehog signalling) is an inhibitor, PSENEN also cleaves the shh ligand at the cell membrane and Gli3 (GLI family zinc finger 3) is a key DNA binding transcription factor of the shh pathway. As there were 185 EWAS hits for Gli3 only a sample can been displayed in the table.

Table 19 provides a further list of key genes involved in nephrogenesis. The CCDC genes are coiled-coil domain containing proteins. CCDC170 was noted to be key for renal tubule cell formation as was MYH7 (Myosin heavy chain 7) and EPCAM (epithelial cell adhesion molecule), the bone morphogenetic proteins (BMP’s) and the Wnt’s. Indeed the Schrott data included 191 hits for BMP’s and 203 hits for Wnt family members.

**Table 17:** EWAS Hits from Schrott Dataset for Significantly Differentially Methylated Regions for.Notch Signalling.

Nearest Gene Name	Chromosome Number	Nearest Gene Number	Dependency Status	Functional Annotation	Page	Distance from Nearest Gene	Relative Position	*p*-Value	Bonferroni Adjusted *p*-Value
JAG1	20	ENSG00000101384	Dependence	Notch Ligand	36	0	Intron	1.31 × 10^−6^	0.007992
NOTCH2	1	ENSG00000134250	Dependence	Notch Pathway	55	0	Intron	3.07 × 10^−6^	0.007992
NOTCH1	9	ENSG00000101384	Dependence	Notch Pathway	56	0	Intron	3.29 × 10^−6^	0.012443
NOTCH2	1	ENSG00000134250	Dependence	Notch Pathway	64	0	Intron	4.19 × 10^−6^	0.013994
NOTCH3	19	ENSG00000074181	Dependence	Notch Pathway	86	3797	Downstream	7.65 × 10^−6^	0.018677
NOTCH2NLC	1	ENSG00000286219	Withdrawal	Notch Pathway	165	0	Intron	2.85 × 10^−6^	0.012636
RBPJ	4	ENSG00000168214	Dependence	Notch Pathway	107	0	Intron	1.17 × 10^−5^	0.022698
RBPJP2	9	ENSG00000274181	Dependence	Notch Pathway	121	19867	Upstream	1.50 × 10^−5^	0.025700
RBPJ	4	ENSG00000168214	Withdrawal	Notch Pathway	125	0	Intron	1.34 × 10^−8^	0.000859
**Nearest Gene Name**	**Chromosome Number**	**Nearest Gene Number**	**Dependency Status**	**Functional Annotation**	**Page**	**Function**	**Number Genes Identified**	***p*-Value**	
PSENEN	19	ENSG00000205155	Withdrawal	Skin Lesion	325	Notch Processing	115	1.65 × 10^−6^	
PSENEN	19	ENSG00000205155	Withdrawal	Skin cancer	325	Notch Processing	113	4.79 × 10^−6^	
PSENEN	19	ENSG00000205155	Withdrawal	Cutaneous melanoma	326	Notch Processing	110	7.71 × 10^−6^	
PSENEN	19	ENSG00000205155	Withdrawal	Solid Organ cancer	327	Notch Processing	150	9.16 × 10^−6^	
PSENEN	19	ENSG00000205155	Withdrawal	Cancer	329	Notch Processing	151	4.32 × 10^−5^	
PSENEN	19	ENSG00000205155	Withdrawal	Melanoma	329	Notch Processing	115	6.21 × 10^−5^	
PSENEN	19	ENSG00000205155	Withdrawal	Solid Organ cancer	335	Notch Processing	149	1.63 × 10^−4^	
PSENEN	19	ENSG00000205155	Withdrawal	Protein processing	353	Notch Processing	4	4.69 × 10^−3^	
PSENEN	19	ENSG00000205155	Withdrawal	Organismal death	356	Notch Processing	39	6.70 × 10^−3^	

**Table 18:** EWAS Hits from Schrott Dataset for Significantly Differentially Methylated Regions for. Sonic Hedgehog Signalling.

Nearest Gene Name	Chromosome Number	Nearest Gene Number	Dependency Status	Functional Annotation	Page	Distance from Nearest Gene	Relative Position	*p*-Value	Bonferroni Adjusted *p*-Value
PTCH1	9	ENSG00000185920	Dependence	Shh Receptor	58	0	Intron	3.46 × 10^−6^	0.012789
PTCHD1-AS	X	ENSG00000233067	Dependence	lnc Promoter/enhancer	91	0	Intron	8.61 × 10^−6^	0.019678
PTCHD1-AS	X	ENSG00000233067	Withdrawal	lnc Promoter/enhancer	129	0	Intron	8.21 × 10^−8^	0.002096
PTCHD4	6	ENSG00000244694	Withdrawal	Shh Receptor; Otopalatodigital syndrome	138	0	Intron	4.21 × 10^−7^	0.005104
PTCH1	9	ENSG00000185920	Withdrawal	Shh Receptor	185	0	Intron	5.80 × 10^−6^	0.017679
SUFU	16	ENSG00000161996	Withdrawal	Hedgehog Inhibitor	207	0	Exon	1.01 × 10^−5^	0.022942
Gli3	7	ENSG00000106571	Dependence	Shh mediator	78	81,232	Downstream	6.35 × 10^−6^	0.017090
Gli3	7	ENSG00000106571	Dependence	Shh mediator	99	0	Intron	1.00 × 10^−5^	0.021181
Gli3	7	ENSG00000106571	Withdrawal	Shh mediator	124	20,318	Downstream	8.23 × 10^−9^	0.000646
Gli3	7	ENSG00000106571	Withdrawal	Shh mediator	182	0	Intron	5.28 × 10^−6^	0.001687
Gli3	7	ENSG00000106571	Withdrawal	Shh mediator	231	0	Intron	1.62 × 10^−5^	0.028539
**Nearest Gene Name**	**Chromosome Number**	**Nearest Gene Number**	**Dependency Status**	**Functional Annotation**	**Page**	**Function**	**Number Genes Identified**	***p*-Value**	
PTCH1	9	ENSG00000185920	KEGG Pathway	Notch Processing	237	Notch Processing	31	0.044117	
PTCH1	9	ENSG00000185920	KEGG Pathway	Skin cancer	238	Notch Processing	54	0.067770	
PSENEN	19	ENSG00000185920	Withdrawal	Cutaneous melanoma	326	Notch Processing	110	0.000008	
Gli3	7	ENSG00000106571	Withdrawal	Skin lesion	325	Notch TF	115	1.65 × 10^−6^	
Gli3	7	ENSG00000106571	Withdrawal	Head & Neck SCC	325	Notch TF	53	3.59 × 10^−6^	
Gli3	7	ENSG00000106571	Withdrawal	Skin cancer	325	Notch TF	113	4.79 × 10^−6^	
Gli3	7	ENSG00000106571	Withdrawal	Lung adenocarcinoma	325	Notch TF	42	5.84 × 10^−6^	
Gli3	7	ENSG00000106571	Withdrawal	Cancer	325	Notch TF	149	7.17 × 10^−6^	
Gli3	7	ENSG00000106571	Withdrawal	Large bowel cancer	326	Notch TF	120	7.45 × 10^−6^	
Gli3	7	ENSG00000106571	Withdrawal	Cutaneous melanoma	326	Notch TF	110	7.71 × 10^−6^	
Gli3	7	ENSG00000106571	Withdrawal	Highg grade astrocytoma	326	Notch TF	82	8.42 × 10^−6^	
Gli3	7	ENSG00000106571	Withdrawal	Abdominal adenocarcinoma	326	Notch TF	135	8.46 × 10^−6^	
Gli3	7	ENSG00000106571	Withdrawal	Solid cancer	327	Notch TF	150	9.16 × 10^−6^	
Gli3	7	ENSG00000106571	Withdrawal	Haed and Neck cancer	327	Notch TF	137	9.54 × 10^−6^	
Gli3	7	ENSG00000106571	Withdrawal	Sensory development	327	Notch TF	18	1.30 × 10^−5^	
Gli3	7	ENSG00000106571	Withdrawal	Carcinoma	327	Notch TF	148	1.38 × 10^−5^	

**Table 19:** EWAS Hits from Schrott Dataset for Significantly Differentially Methylated Regions for. Major Genes of Nephrogenesis I.

Nearest Gene Name	Chromosome Number	Nearest Gene Number	Dependency Status	Functional Annotation	Page	Distance from Nearest Gene	Relative Position	*p*-Value	Bonferroni Adjusted *p*-Value
CCDC178	18	ENSG00000166960	Dependence	Coiled coil domain containing	21	0	Intron	3.78 × 10^−7^	0.004344
CCDC178	18	ENSG00000166960	Dependence	Coiled coil domain containing	47	0	Intron	2.29 × 10^−6^	0.010512
CCDC171	9	ENSG00000164989	Dependence	Coiled coil domain containing	53	0	Intron	2.88 × 10^−6^	0.011635
CCDC178	18	ENSG00000166960	Dependence	Coiled coil domain containing	119	0	Intron	1.46 × 10^−5^	0.025394
CCDC171	9	ENSG00000164989	Withdrawal	Coiled coil domain containing	359	0	Intron	8.36 × 10^−8^	0.002109
CCDC170	6	ENSG00000120262	Withdrawal	Coiled coil domain containing	136	0	Intron	3.29 × 10^−7^	0.004420
CCDC175	14	ENSG00000151838	Withdrawal	Coiled coil domain containing	148	0	Intron	1.11 × 10^−6^	0.008146
CCDC171	9	ENSG00000164989	Withdrawal	Coiled coil domain containing	169	57,410	Downstream	3.38 × 10^−6^	0.013764
CCDC171	9	ENSG00000164989	Withdrawal	Coiled coil domain containing	170	0	Intron	3.50 × 10^−6^	0.139199
CCDC178	18	ENSG00000166960	Withdrawal	Coiled coil domain containing	186	0	Intron	5.94 × 10^−6^	0.017905
CCDC171	9	ENSG00000164989	Withdrawal	Coiled coil domain containing	189	0	Intron	6.67 × 10^−6^	0.019063
CCDC171	9	ENSG00000164989	Withdrawal	Coiled coil domain containing	203	0	Intron	9.26 × 10^−6^	0.022131
MYH7	14	ENSG00000092054	Dependence	Myosin heavy chain 7B	81	12,605	Upstream	6.91 × 10^−6^	0.017781
ROCK1	18	ENSG00000067900	Dependence	Rho associated Coiled Coil Kinase	20	2,527,461	Downstream	3.17 × 10^−7^	0.003991
ROCK1	18	ENSG00000067900	Dependence	Rho associated Coiled Coil Kinase	20	0	Intron	5.41 × 10^−6^	0.015884
ROCK1	18	ENSG00000067900	Dependence	Rho associated Coiled Coil Kinase	20	411,279	Downstream	4.72 × 10^−6^	0.016044
EPCAM-DT	2	ENSG00000234690	Dependence	Epithelial Cell adhesion molecule—divergent transcript	221	0	Intron	1.33 × 10^−5^	0.025950
BMPR2	1	ENSG00000204217	Dependence	BMP Receptor 2	12	0	Intron	8.38 × 10^−8^	0.001951
BMPR1B	4	ENSG00000138696	Dependence	BMP Receptor 1B	25	0	Intron	5.68 × 10^−7^	0.005306
BMPR2	4	ENSG00000138696	Dependence	BMP Receptor 1B	35	0	Intron	1.22 × 10^−6^	0.007711
BMPER	7	ENSG00000164619	Dependence	BMP Endothelial regulator	42	0	Intron	1.83 × 10^−6^	0.009422
BMP6	6	ENSG00000153162	Dependence	BMP 6	56	0	Intron	3.18 × 10^−6^	0.012217
BMPR1B	4	ENSG00000138696	Dependence	BMP Receptor 1B	70	0	Intron	5.19 × 10^−6^	0.015541
BMPR1A	10	ENSG00000107779	Dependence	BMP Receptor 1A	96	0	Intron	9.50 × 10^−6^	0.020621
BMPR1B	4	ENSG00000138696	Dependence	BMP Receptor 1B	98	0	Intron	9.94 × 10^−6^	0.021066
BMPER	7	ENSG00000164619	Dependence	BMP Endothelial regulator	113	0	Intron	1.32 × 10^−5^	0.024145
BMPR1B	4	ENSG00000138696	Withdrawal	BMP Receptor 1B	124	0	Intron	7.92 × 10^−9^	0.000631
WNT7B	22	ENSG00000188064	Dependence	Wnt family member 7B	74	0	Intron	5.78 × 10^−6^	0.016375
WNT7B	22	ENSG00000188064	Withdrawal	Wnt family member 7B	161	0	Intron	2.38 × 10^−6^	0.011749
WNT7A	3	ENSG00000154764	Dependence	Wnt family member 7A	119	0	Intron	1.47 × 10^−5^	0.025468
WNT7A	3	ENSG00000154764	Withdrawal	Wnt family member 7A	123	0	Intron	4.13 × 10^−9^	0.000453
WNT2	7	ENSG00000105989	Dependence	Wnt family member 2	26	0	Intron	1.02 × 10^−6^	0.007849
WNT5A	3	ENSG00000114251	Withdrawal	Wnt family member 5A	150	26,665	Downstream	1.25 × 10^−6^	0.008600
WNT7A	3	ENSG00000154764	Dependence	Wnt family member 7A	150	13,677	Upstream	1.27 × 10^−6^	0.008678
WNT7B	22	ENSG00000188064	Withdrawal	Wnt family member 7B	161	0	Intron	2.38 × 10^−6^	0.011749
WNT9B	17	ENSG00000158955	Withdrawal	Wnt family member 9B	211	0	Intron	1.10 × 10^−5^	0.023817
WNT16	7	ENSG00000002745	Withdrawal	Wnt family member 16	227	0	Intron	1.50 × 10^−5^	0.027474

Some of the hits for the Wnt’s are listed in Table 20 and their very high level of statistical significance is evident.

Table 21 lists some of the Schrott EWAS hits for ROCK1.

TGFβ was also shown to be important in the renal organoid GWAS screen and induced interstitial fibrosis in line with its well known activities in many other tissues. Eight EWAS hits from the Schrott data for TGFβ are shown in Table 22. Downstream TGFβ signals are transduced by a cascade of SMAD (mothers against decapentaplegic homologue) intermediates and transcription factors. SMAD4 is the final transcription factor to enter the nucleus from this pathway. Cannabis exposure EWAS hits for this series of mediators is also shown in the table. 

### 4.10. Exponential Genotoxic Effects

A wide variety of studies have documented the exponential dose–response relationship of mutagenicity and various indices of cannabinoids exposure [43,65,66,67,68,69,70,71,72,73,74,75,76,77,78]. This applies both to directly genotoxic studies and to studies of the intermediary mitochondrial metabolism upon which genomic and epigenomic reactions are based both as substrates and for energy supply. Moreover, several reports confirm that the exponential effects seen in the laboratory are also documented in patterns of human disease, particularly by modelling studies and by showing an abrupt discontinuous step in disease rates from the fourth to the faith quintile of cannabinoid exposure.

This exponential cannabinoid dose–response relationship where disease rates occur at higher levels of cannabinoid exposure is very important when interpreting epidemiological patterns of UCAR’s. This is especially so for Europe where increases in cannabis use prevalence, cannabis use daily intensity, and THC concentration in cannabis herb and resin are all going on at the same time creating an exponential cannabinoid exposure curve. This in turn implies that whole nations can be relatively suddenly moved up into the genotoxic dose–response zone where major and severe genotoxic outcomes become commonplace. As was noted this may account for severe and apparently abrupt teratological outbreaks such as the limblessness phenomena described earlier.

### 4.11. Strengths and Limitations

This study has many strengths including the use of one of the largest UCAR databases in the world and the very comprehensive EMCDDA drug dataset. Moreover, advanced statistical modelling has been utilized in performing this analysis including inverse probability weighting, lagged models, the formal tools of quantitative causal inference and geospatiotemporal regression modelling. Panelled maps and graphs have been used to show all covariates across time at a single glace. Bivariate maps have also been used, which is unusual. Moreover, a sophisticate mechanistic understanding has been presented which not only explains the pattern of UCAR’s observed but contributes pivotal information into the key issue of causal assignment. Study limitations include the lack of individual participant exposure information, a limitation which is shared in common with many epidemiological investigations. Moreover, missing data was an issue form some covariates particularly daily cannabis use and this must be borne in mind in considering the present results.

### 4.12. Generalizability

Results of this study are widely generalizable for several reasons. The analysis is based on one of the largest UCAR datasets in the world, and concordant with similar published results presented from other jurisdictions. Further, results are at high levels statistical significance and sensitivity analysis using E-values indicates that they are robust to extraneous explanations. Moreover, the analysis has been conducted within a formal quantitative causal inferential framework so that the effects demonstrated may be designated as causal effects. For all of these reasons we feel that these results are widely generalizable to other situations wherever data of sufficient quality exist to make the relevant assessments.

## 5. Conclusions

In conclusion this analysis confirms previous reports showing that many UCA’s are related to various metrics of cannabis exposure. To those anomalies which have already been described this report adds congenital hydronephrosis, multicystic renal disease and urinary anomalies overall as newly cannabis associated congenital anomalies. This analysis has been conducted within a causal inferential framework including the use of inverse probability weighting and E-values which formally demonstrate that the quantitative criteria of formal causal inference are fulfilled. Particular concern applies to the relatively rapid rise of many parts of Europe into the higher dose cannabinoid exposure zone including by contamination of the food chain where major genotoxic outcomes become commonplace. As discussed there is some evidence that this may be occurring in parts of France and Germany already. Another major and related corollary is real concern for multigenerational damage to the epigenome. Evidence presented indicates that epigenomic explanations may well underlie many of the effects observed and the potential for transgenerational transmission of epigenomic effects is very real and of great concern. Such issues, together with the robust and causal relationships demonstrated in the present investigation indicate that community cannabinoid penetration should be carefully and closely constrained and restricted and appropriate protections must be put in place for protection of the food chain and the genomes and epigenomes of generations yet to come.

## Figures and Tables

**Table 10:** E-Value List.

No.	E-Value Estimate	Lower Bound E-Value
1	3.41 × 10^122^	3.30 × 10^81^
2	1.18 × 10^103^	1.36 × 10^31^
3	1.45 × 10^91^	1.08 × 10^30^
4	5.23 × 10^72^	1.67 × 10^26^
5	6.01 × 10^67^	4.98 × 10^23^
6	6.23 × 10^63^	1.24 × 10^17^
7	3.34 × 10^47^	1.14 × 10^10^
8	5.81 × 10^28^	1.39 × 10^9^
9	4.09 × 10^18^	1.95 × 10^8^
10	5.96 × 10^17^	2.01 × 10^6^
11	1.21 × 10^17^	1.22 × 10^5^
12	2.18 × 10^13^	1.15 × 10^5^
13	3.51 × 10^9^	3.52 × 10^4^
14	6.69 × 10^8^	2.03 × 10^4^
15	4.51 × 10^8^	1.46 × 10^3^
16	2.33 × 10^7^	1258.11
17	6.89 × 10^6^	303.56
18	2.74 × 10^5^	285.07
19	1.61 × 10^5^	119.49
20	1.23 × 10^5^	83.68
21	8.150 × 10^4^	83.66
22	6.62 × 10^4^	56.63
23	2.73 × 10^4^	43.52
24	6.62 × 10^3^	43.51
25	3540.00	22.36
26	955.32	17.85
27	954.95	13.74
28	606.01	13.62
29	365.38	9.43
30	273.00	9.28
31	252.91	9.09
32	162.57	8.35
33	161.31	7.02
34	82.56	5.47
35	67.00	5.45
36	59.95	5.18
37	43.56	4.73
38	42.81	3.75
39	21.28	3.38
40	15.85	3.23
41	11.7	3.13
42	10.52	3.06
43	9.68	2.51
44	9.68	2.38
45	9.03	2.10
46	8.58	2.02
47	7.24	2.02
48	6.58	1.89
49	5.01	1.62
50	2.86	1.60
51	2.65	1.60

**Table 16:** EWAS Hits from Schrott Dataset for Significantly Differentially Methylated Regions for.Uronephrological Congenital Anomalies.

Functional Annotation	Status	Page	Number Genes Identified	*p*-Value
**Congenital Anomalies**				
Congenital anomalies of kidney and urinary tract	Withdrawal	351	9	0.003850
Familial congenital anomalies of kidney and urinary tract	Withdrawal	352	8	0.004400
Urological diseases	Withdrawal	357	1	0.007010
Renal agenesis	Withdrawal	311	5	0.000429
Renal tubular morphology	Withdrawal	313	8	0.000606
Renal morphology & development	Withdrawal	322	16	0.002160
Morphology Urinary system	Withdrawal	322	17	0.002200
Renal lesions	Withdrawal	322	48	0.002200
Renal tubular abnormality	Withdrawal	323	7	0.002550
Urinary tract cancer	Withdrawal	320	84	0.001890
**Male Cancers**				
Male genital neoplasm	Withdrawal	341	60	0.000675
Male genital neoplasm	Dependence	279	153	1.14 × 10^−8^
Malignant male genital neoplasm	Dependence	279	151	1.60 × 10^−8^
Prostate cancer	Dependence	279	148	2.88 × 10^−8^
Prostate cancer	Withdrawal	340	59	5.33 × 10^−4^
Prostate cancer cell adhesion	Withdrawal	357	1	0.007010
**Female Cancers**				
Female genital neoplasm	Withdrawal	349	78	0.003030
Female genital neoplasm	Withdrawal	350	79	0.003460
Female genital adenocarcinoma	Dependence	272	197	1.84 × 10^−11^
Female genital carcinoma	Dependence	273	215	2.05 × 10^−11^
Female genital carcinoma	Dependence	276	276	8.95 × 10^−11^

**Table 20:** EWAS Hits from Schrott Dataset for Significantly Differentially Methylated Regions for. Major Genes of Nephrogenesis II—Wnt’s.

Nearest Gene Name	Page	Functional Annotation	Number Genes Identified	*p*-Value
WNT3A	239	Head and neck cancer	356	7.73 × 10^−20^
WNT8B	239	Head and neck cancer	356	7.73 × 10^−20^
WNT3A	239	Head and neck cancer	342	7.74 × 10^−20^
WNT8B	239	Head and neck cancer	342	7.74 × 10^−20^
WNT3A	240	Head and neck cancer	333	3.34 × 10^−19^
WNT8B	240	Head and neck cancer	333	3.34 × 10^−19^
WNT3A	240	Abdominal carcinoma	362	3.64 × 10^−19^
WNT8B	240	Abdominal carcinoma	362	3.64 × 10^−19^
WNT3A	241	Solid cancer	381	6.93 × 10^−18^
WNT8B	241	Solid cancer	381	6.93 × 10^−18^
WNT3A	241	Thyroid cancer	318	1.21 × 10^−17^
WNT8B	241	Thyroid cancer	318	1.21 × 10^−17^
WNT3A	242	Thyroid cancer	319	1.26 × 10^−17^
WNT8B	242	Thyroid cancer	319	1.26 × 10^−17^
WNT3A	242	Endocrine tumour	321	1.44 × 10^−17^
WNT8B	242	Endocrine tumour	321	1.44 × 10^−17^

**Table 21:** EWAS Hits from Schrott Dataset for Significantly Differentially Methylated Regions for. Major Genes of Nephrogenesis III—ROCK1.

Nearest Gene Name	Page	Functional Annotation	Number Genes Identified	*p*-Value	Bonferroni Adjusted *p*-Value
ROCK1	236	Focal Adhesion	39	0.000934	0.226633
ROCK1	236	Vascular Smooth Muscle	25	0.001862	0.400987
ROCK1	236	cGMP-PKG signalling	30	0.003895	0.658065
ROCK1	237	Actin Cytoskeleton	37	0.004587	0.717564
ROCK1	237	Sphingolipid signalling	24	0.005596	0.786305
ROCK1	237	Axon guidance	24	0.011178	0.954555
ROCK1	237	Oxytocin signalling	25	0.039031	1.000000
ROCK1	237	TGFβ signalling	16	0.040247	1.000000
ROCK1	237	cAMP signalling	31	0.044117	1.000000
ROCK1	237	Leukocyte transendothelial migration	20	0.046734	1.000000
ROCK1	238	Cancer pathways	54	0.067770	1.000000
ROCK1	238	Platelet activation	21	0.077866	1.000000

**Table 22:** EWAS Hits from Schrott Dataset for Significantly Differentially Methylated Regions. For TGFβ—Signalling.

Nearest Gene Name	Page	Status	Functional Annotation	Number Genes Identified	*p*-Value	Bonferroni Adjusted *p*-Value
**TGFβ**						
TGFB1	50	Dependence	Fibrosis		2.53 × 10^−6^	0.011012
TGFB1	237		Chronic myeloid leukaemia	16	7.73 × 10^−20^	0.095040
TGFB1	237		Colorectal cancer	14	0.004626	0.988843
TGFB1	237		Hippo signalling	26	0.004047	0.998936
TGFB1	237		MAPK pathway	39	0.007516	1.000000
TGFB1	237		TGFβ signalling	16	0.040246	1.000000
TGFB1	238		Cancer pathways	54	0.067770	1.000000
TGFB1	238		Amoebiasis	18	0.072579	1.000000
**Nearest Gene Name**	**Page**	**Status**	**Gene Number**	**Chromosome Number**	***p*-Value**	**Bonferroni Adjusted *p*-Value**
**SMADs**						
SMAD2	6	Dependence	ENSG00000175387	18	9.32 × 10^−9^	0.000632
SMAD7	26	Dependence	ENSG00000101665	18	6.18 × 10^−7^	0.005518
SMAD2	53	Dependence	ENSG00000175387	18	2.90 × 10^−6^	0.011688
SMAD9	85	Dependence	ENSG00000120693	13	7.47 × 10^−6^	0.018446
SMAD6	125	Withdrawal	ENSG00000137834	15	2.26 × 10^−8^	0.001125
SMAD3	166	Withdrawal	ENSG00000166949	15	2.96 × 10^−6^	0.012904
SMAD7	169	Withdrawal	ENSG00000101665	18	3.40 × 10^−6^	0.013798
SMAD2	184	Withdrawal	ENSG00000175387	18	5.65 × 10^−6^	0.017449
SMAD3	209	Withdrawal	ENSG00000166949	15	1.07 × 10^−5^	0.023594
SMAD2	236		ENSG00000175387	18	0.003911	

## Data Availability

All data generated or analysed during this study are included in this published article and its Supplementary Information Files. Data along with the relevant R code has been made publicly available on the Mendeley Database Repository and can be accessed from these URL’s: https://data.mendeley.com/datasets/c6psrbr34j/2 (accessed on 1 February 2022) and https://data.mendeley.com/datasets/vd6mt5r5jm/1 (accessed on 1 February 2022).

## Supplementary Materials

The following supporting information can be downloaded at: https://www.mdpi.com/article/10.3390/ijerph192113769/s1, Figure S1: Geospatial Links; Table S1: Overall Study Profile; Table S2, Daily Cannabis Use—Raw Data; Table S3: Daily Cannabis Use—Interpolated Data; Table S4: All regression slopes and results from bivariate analyses; Table S5: Variable Importance Tables from Ranger random forest regression—Uronephrological anomalies; Table S6: Variable Importance Tables from Ranger random forest regression—Multicystic renal disease; Table S7: Variable Importance Tables from Ranger random forest regression—Bilateral renal agenesis; Table S8: Variable Importance Tables from Ranger random forest regression—Hydronephrosis; Table S9: Variable Importance Tables from Ranger random forest regression—Congenital posterior urethral valve; Table S10: Inverse probability weighted panel regression results—Uronephrological anomalies; Table S11: Inverse probability weighted panel regression results—Multicystic renal disease; Table S12: Inverse probability weighted panel regression results—Bilateral renal agenesis; Table S13: Inverse probability weighted panel regression results—Hydronephrosis; Table S14: Inverse probability weighted panel regression results—Congenital posterior urethral valve.

## References

[1] Reece A.S., Hulse G.K. (2022). Geotemporospatial and Causal Inferential Epidemiological Overview and Survey of USA Cannabis, Cannabidiol and Cannabinoid Genotoxicity Expressed in Cancer Incidence 2003–2017: Part 1–Continuous Bivariate Analysis. Arch. Public Health.

[2] Reece A.S., Hulse G.K. (2022). Geotemporospatial and Causal Inferential Epidemiological Overview and Survey of USA Cannabis, Cannabidiol and Cannabinoid Genotoxicity Expressed in Cancer Incidence 2003–2017: Part 2–Categorical Bivariate Analysis and Attributable Fractions. Arch. Public Health.

[3] Reece A.S., Hulse G.K. (2022). Geotemporospatial and Causal Inferential Epidemiological Overview and Survey of USA Cannabis, Cannabidiol and Cannabinoid Genotoxicity Expressed in Cancer Incidence 2003–2017: Part 3–Spatiotemporal, Multivariable and Causal Inferential Pathfinding and Exploratory Analyses of Prostate and Ovarian Cancers. Arch. Public Health.

[4] Reece A.S., Hulse G.K. (2021). Cannabinoid exposure as a major driver of pediatric acute lymphoid Leukaemia rates across the USA: Combined geospatial, multiple imputation and causal inference study. BMC Cancer.

[5] Reece A.S., Hulse G.K. (2021). A geospatiotemporal and causal inference epidemiological exploration of substance and cannabinoid exposure as drivers of rising US pediatric cancer rates. BMC Cancer.

[6] Reece A.S., Hulse G.K. (2020). Contemporary epidemiology of rising atrial septal defect trends across USA 1991-2016: A combined ecological geospatiotemporal and causal inferential study. BMC Pediatrics.

[7] Reece A.S., Hulse G.K. (2022). Geotemporospatial and causal inference epidemiological analysis of US survey and overview of cannabis, cannabidiol and cannabinoid genotoxicity in relation to congenital anomalies 2001–2015. BMC Pediatrics.

[8] Reece A.S., Hulse G.K. (2020). Broad Spectrum epidemiological contribution of cannabis and other substances to the teratological profile of northern New South Wales: Geospatial and causal inference analysis. BMC Pharmacol. Toxicol..

[9] Reece A.S., Hulse G.K. (2021). Causal inference multiple imputation investigation of the impact of cannabinoids and other substances on ethnic differentials in US testicular cancer incidence. BMC Pharmacol. Toxicol..

[10] Reece A.S., Hulse G.K. (2020). Co-occurrence across time and space of drug- and cannabinoid- exposure and adverse mental health outcomes in the National Survey of Drug Use and Health: Combined geotemporospatial and causal inference analysis. BMC Public Health.

[11] Reece A.S., Hulse G.K., Preedy V., Patel V. (2022). Cannabis Genotoxicity and Cancer Incidence: A Highly Concordant Synthesis of European and USA Datasets. Cannabis, Cannabinoids and Endocannabinoids. Volume 1.

[12] Reece A.S., Hulse G.K., Preedy V., Patel V. (2022). Cannabinoid Genotoxicity and Congenital Anomalies: A Convergent Synthesis of European and USA Datasets. Cannabis, Cannabinoids and Endocannabinoids. Volume 1.

[13] Reece A.S., Hulse G.K. (2019). Cannabis Teratology Explains Current Patterns of Coloradan Congenital Defects: The Contribution of Increased Cannabinoid Exposure to Rising Teratological Trends. Clin. Pediatrics.

[14] Reece A.S., Hulse G.K. (2019). Epidemiological Associations of Various Substances and Multiple Cannabinoids with Autism in USA. Clin. Pediatrics Open Access.

[15] Reece A.S., Hulse G.K. (2022). Cannabinoid- and Substance- Relationships of European Congenital Anomaly Patterns: A Space-Time Panel Regression and Causal Inferential Study. Environ. Epigenetics.

[16] Reece A.S., Hulse G.K. (2022). Geospatiotemporal and Causal Inference Study of Cannabis and Other Drugs as Risk Factors for Female Breast Cancer USA 2003-2017. Environ. Epigenetics.

[17] Reece A.S., Hulse G.K. (2019). Cannabis Consumption Patterns Explain the East-West Gradient in Canadian Neural Tube Defect Incidence: An Ecological Study. Glob. Pediatrics Health.

[18] Reece A.S., Hulse G.K. (2020). Canadian Cannabis Consumption and Patterns of Congenital Anomalies: An Ecological Geospatial Analysis. J. Addict. Med..

[19] Forrester M.B., Merz R.D. (2007). Risk of selected birth defects with prenatal illicit drug use, Hawaii, 1986–2002. J. Toxicol. Environ. Health.

[20] Reece A.S., Hulse G.K. (2021). Quadruple convergence—Rising cannabis prevalence, intensity, concentration and use disorder treatment. Lancet Reg. Health Eur..

[21] Reece A.S., Hulse G.K. (2022). Epidemiological Overview of Cannabis- and Substance- Carcinogenesis in Europe: A Lagged Causal Inferential Panel Regression Modelling and Marginal Effects Study.

[22] Reece A.S., Hulse G.K. (2020). Cannabis in Pregnancy—Rejoinder, Exposition and Cautionary Tales. Psychiatr. Times.

[23] Reece A.S., Hulse G.K. (2021). Epidemiological Overview of Multidimensional Chromosomal and Genome Toxicity of Cannabis Exposure in Congenital Anomalies and Cancer Development. Sci. Rep..

[24] Reece A.S. (2022). Rapid Response: Cannabinoid Genotoxic Trifecta—Cancerogenesis, Clinical Teratogenesis and Cellular Ageing. Br. Med. J..

[25] Reece A.S. (2022). Limblessness: Cannabinoids Inhibit Key Embryonic Morphogens both Directly and Epigenomically. Br. Med. J..

[26] Reece A.S., Hulse G.K. Geospatiotemporal and Causal Inferential Epidemiological Survey and Exploration of Cannabinoid- and Substance- Related Carcinogenesis in USA 2003–2017. Epidemiology of Cannabis: Genotoxicity and Neurotoxicity, Epigenomics and Aging.

[27] Reece A.S., Hulse G.K. (2022). European Epidemiological Patterns of Cannabis- and Substance- Related Congenital Body Wall Anomalies: Geospatiotemporal and Causal Inferential Study. Intern. J. Environ. Res. Public Health.

[28] Reece A.S., Hulse G.K. (2022). Cannabis- and Substance- Related Epidemiological Patterns of Chromosomal Congenital Anomalies in Europe: Geospatiotemporal and Causal Inferential Study. Int. J. Environ. Res. Public Health.

[29] Reece A.S., Hulse G.K. (2022). State Trends of Cannabis Liberalization as a Causal Driver of Increasing Testicular Cancer Rates across the USA. Int. J. Environ. Res. Public Health.

[30] Reece A.S., Hulse G.K. (2022). Epidemiology of Δ8THC-Related Carcinogenesis in USA: A Panel Regression and Causal Inferential Study. Int. J. Environ. Res. Public Health.

[31] Reece A.S., Hulse G.K. (2022). Epidemiological association of cannabinoid- and drug- exposures and sociodemographic factors with limb reduction defects across USA 1989–2016: A geotemporospatial study. Spat. Spatio-Temporal Epidemiol..

[32] Manthey J., Freeman T.P., Kilian C., Lopez-Pelayo H., Rehm J. (2021). Public health monitoring of cannabis use in Europe: Prevalence of use, cannabis potency, and treatment rates. Lancet Reg. Health Eur..

[33] Reece A.S., Hulse G.K. (2022). Epigenomic and Other Evidence for Cannabis-Induced Aging Contextualized in a Synthetic Epidemiologic Overview of Cannabinoid-Related Teratogenesis and Cannabinoid-Related Carcinogenesis. Mendeley Data.

[34] Reece A.S., Norman A., Hulse G.K. (2016). Cannabis exposure as an interactive cardiovascular risk factor and accelerant of organismal ageing: A longitudinal study. BMJ Open.

[35] Reece A.S., Hulse G.K. Extending the “Paracentral Dogma” of biology with the metabolome: Implications for understanding genomic-glycomic-metabolic-epigenomic synchronization. Engineering.

[36] Reece A.S., Hulse G.K. Cannabis, Cannabidiol, Cannabinoids and Multigenerational Policy. Engineering.

[37] Huang H.F.S., Nahas G.G., Hembree W.C., Nahas G.G., Sutin K.M., Harvey D.J., Agurell S. (1999). Effects of Marijuana Inhalation on Spermatogenesis of the Rat. Marijuana in Medicine.

[38] Zimmerman A.M., Zimmerman S., Raj A.Y., Nahas G.G., Sutin K.M., Harvey D.J., Agurell S. (1999). Effects of Cannabinoids on Spermatogensis in Mice. Marijuana and Medicine.

[39] Morishima A. (1984). Effects of cannabis and natural cannabinoids on chromosomes and ova. NIDA Res. Monogr..

[40] Rossato M., Ion Popa F., Ferigo M., Clari G., Foresta C. (2005). Human sperm express cannabinoid receptor Cb1, the activation of which inhibits motility, acrosome reaction, and mitochondrial function. J. Clin. Endocrinol. Metab..

[41] Rossato M., Pagano C., Vettor R. (2008). The cannabinoid system and male reproductive functions. J. Neuroendocrinol..

[42] Chioccarelli T., Cacciola G., Altucci L., Lewis S.E., Simon L., Ricci G., Ledent C., Meccariello R., Fasano S., Pierantoni R. (2010). Cannabinoid receptor 1 influences chromatin remodeling in mouse spermatids by affecting content of transition protein 2 mRNA and histone displacement. Endocrinology.

[43] Russo C., Ferk F., Mišík M., Ropek N., Nersesyan A., Mejri D., Holzmann K., Lavorgna M., Isidori M., Knasmüller S. (2019). Low doses of widely consumed cannabinoids (cannabidiol and cannabidivarin) cause DNA damage and chromosomal aberrations in human-derived cells. Arch. Toxicol..

[44] Stenchever M.A., Kunysz T.J., Allen M.A. (1974). Chromosome breakage in users of marihuana. Am. J. Obstet. Gynecol..

[45] Leuchtenberger C., Leuchtenberger R. (1971). Morphological and cytochemical effects of marijuana cigarette smoke on epithelioid cells of lung explants from mice. Nature.

[46] McClintock B. (1938). The Production of Homozygous Deficient Tissues with Mutant Characteristics by Means of the Aberrant Mitotic Behavior of Ring-Shaped Chromosomes. Genetics.

[47] Shen H., Shih J., Hollern D.P., Wang L., Bowlby R., Tickoo S.K., Thorsson V., Mungall A.J., Newton Y., Hegde A.M. (2018). Integrated Molecular Characterization of Testicular Germ Cell Tumors. Cell Rep..

[48] DiNieri J.A., Wang X., Szutorisz H., Spano S.M., Kaur J., Casaccia P., Dow-Edwards D., Hurd Y.L. (2011). Maternal cannabis use alters ventral striatal dopamine D2 gene regulation in the offspring. Biol. Psychiatry.

[49] Szutorisz H., DiNieri J.A., Sweet E., Egervari G., Michaelides M., Carter J.M., Ren Y., Miller M.L., Blitzer R.D., Hurd Y.L. (2014). Parental THC exposure leads to compulsive heroin-seeking and altered striatal synaptic plasticity in the subsequent generation. Neuropsychopharmacology.

[50] Watson C.T., Szutorisz H., Garg P., Martin Q., Landry J.A., Sharp A.J., Hurd Y.L. (2015). Genome-Wide DNA Methylation Profiling Reveals Epigenetic Changes in the Rat Nucleus Accumbens Associated with Cross-Generational Effects of Adolescent THC Exposure. Neuropsychopharmacology.

[51] Szutorisz H., Hurd Y.L. (2016). Epigenetic Effects of Cannabis Exposure. Biol. Psychiatry.

[52] Szutorisz H., Hurd Y.L. (2018). High times for cannabis: Epigenetic imprint and its legacy on brain and behavior. Neurosci. Biobehav. Rev..

[53] Ellis R.J., Bara A., Vargas C.A., Frick A.L., Loh E., Landry J., Uzamere T.O., Callens J.E., Martin Q., Rajarajan P. (2021). Prenatal Δ(9)-Tetrahydrocannabinol Exposure in Males Leads to Motivational Disturbances Related to Striatal Epigenetic Dysregulation. Biol. Psychiatry.

[54] Murphy S.K., Itchon-Ramos N., Visco Z., Huang Z., Grenier C., Schrott R., Acharya K., Boudreau M.H., Price T.M., Raburn D.J. (2018). Cannabinoid exposure and altered DNA methylation in rat and human sperm. Epigenetics.

[55] Schrott R., Murphy S.K., Modliszewski J.L., King D.E., Hill B., Itchon-Ramos N., Raburn D., Price T., Levin E.D., Vandrey R. (2021). Refraining from use diminishes cannabis-associated epigenetic changes in human sperm. Environ. Epigenetics.

[56] Blevins R.D., Regan J.D. (1976). delta-9-Tetrahydrocannabinol: Effect on macromolecular synthesis in human and other mammalian cells. Arch. Toxicol..

[57] McClean D.K., Zimmerman A.M. (1976). Action of delta 9-tetrahydrocannabinol on cell division and macromolecular synthesis in division-synchronized protozoa. Pharmacology.

[58] Nahas G.G., Morishima A., Desoize B. (1977). Effects of cannabinoids on macromolecular synthesis and replication of cultured lymphocytes. Fed. Proc..

[59] Mon M.J., Jansing R.L., Doggett S., Stein J.L., Stein G.S. (1978). Influence of delta9-tetrahydrocannabinol on cell proliferation and macromolecular biosynthesis in human cells. Biochem. Pharmacol..

[60] Mon M.J., Haas A.E., Stein J.L., Stein G.S. (1981). Influence of psychoactive and nonpsychoactive cannabinoids on cell proliferation and macromolecular biosynthesis in human cells. Biochem. Pharmacol..

[61] Mon M.J., Haas A.E., Stein J.L., Stein G.S. (1981). Influence of psychoactive and nonpsychoactive cannabinoids on chromatin structure and function in human cells. Biochem. Pharmacol..

[62] Yang X., Hegde V.L., Rao R., Zhang J., Nagarkatti P.S., Nagarkatti M. (2014). Histone modifications are associated with Delta9-tetrahydrocannabinol-mediated alterations in antigen-specific T cell responses. J. Biol. Chem..

[63] Mehrnoush V., De Lima S.G., Kotb A., Hyndman M.E. (2022). The association of bladder cancer and Cannabis: A systematic review. Arch. Ital. Urol. Androl..

[64] Payne K.S., Mazur D.J., Hotaling J.M., Pastuszak A.W. (2019). Cannabis and Male Fertility: A Systematic Review. J. Urol..

[65] Busch F.W., Seid D.A., Wei E.T. (1979). Mutagenic activity of marihuana smoke condensates. Cancer Lett..

[66] Zimmerman A.M., Raj A.Y. (1980). Influence of cannabinoids on somatic cells in vivo. Pharmacology.

[67] Tahir S.K., Zimmerman A.M. (1991). Influence of marihuana on cellular structures and biochemical activities. Pharmacol. Biochem. Behav..

[68] Vela G., Martín S., García-Gil L., Crespo J.A., Ruiz-Gayo M., Fernández-Ruiz J.J., Garcia-Lecumberri C., Pelaprat D., Fuentes J.A., Ramos J.A. (1998). Maternal exposure to delta9-tetrahydrocannabinol facilitates morphine self-administration behavior and changes regional binding to central mu opioid receptors in adult offspring female rats. Brain Res..

[69] Koller V.J., Auwarter V., Grummt T., Moosmann B., Misik M., Knasmuller S. (2014). Investigation of the in vitro toxicological properties of the synthetic cannabimimetic drug CP-47,497-C8. Toxicol. Appl. Pharmacol..

[70] Koller V.J., Ferk F., Al-Serori H., Mišík M., Nersesyan A., Auwärter V., Grummt T., Knasmuller S. (2015). Genotoxic properties of representatives of alkylindazoles and aminoalkyl-indoles which are consumed as synthetic cannabinoids. Food Chem. Toxicol..

[71] Price P.J., Suk W.A., Spahn G.J., Freeman A.E. (1972). Transformation of Fischer rat embryo cells by the combined action of murine leukemia virus and (-)-trans- 9 -tetrahydrocannabinol. Proc. Soc. Exp. Biol. Med..

[72] Hölzel B.N., Pfannkuche K., Allner B., Allner H.T., Hescheler J., Derichsweiler D., Hollert H., Schiwy A., Brendt J., Schaffeld M. (2020). Following the adverse outcome pathway from micronucleus to cancer using H2B-eGFP transgenic healthy stem cells. Arch. Toxicol..

[73] Tahir S.K., Trogadis J.E., Stevens J.K., Zimmerman A.M. (1992). Cytoskeletal organization following cannabinoid treatment in undifferentiated and differentiated PC12 cells. Biochem. Cell Biol..

[74] Sarafian T.A., Kouyoumjian S., Khoshaghideh F., Tashkin D.P., Roth M.D. (2003). Delta 9-tetrahydrocannabinol disrupts mitochondrial function and cell energetics. Am. J. Physiol..

[75] Sarafian T.A., Habib N., Oldham M., Seeram N., Lee R.P., Lin L., Tashkin D.P., Roth M.D. (2006). Inhaled marijuana smoke disrupts mitochondrial energetics in pulmonary epithelial cells in vivo. Am. J. Physiol..

[76] Morimoto S., Tanaka Y., Sasaki K., Tanaka H., Fukamizu T., Shoyama Y., Shoyama Y., Taura F. (2007). Identification and characterization of cannabinoids that induce cell death through mitochondrial permeability transition in Cannabis leaf cells. J. Biol. Chem..

[77] Fisar Z., Singh N., Hroudova J. (2014). Cannabinoid-induced changes in respiration of brain mitochondria. Toxicol. Lett..

[78] Singh N., Hroudova J., Fisar Z. (2015). Cannabinoid-Induced Changes in the Activity of Electron Transport Chain Complexes of Brain Mitochondria. J. Mol. Neurosci..

[79] Gant J. (2019). Scientists are baffled by spatter of babies born without hands or arms in France, as investigation fails to discover a cause. Daily Mail.

[80] Agence France-Presse in Paris (2018). France-Presse in Paris. France to investigate cause of upper limb defects in babies. The Guardian.

[81] Willsher K. (2018). Baby arm defects prompt nationwide investigation in France. Guardian.

[82] Babies Born with Deformed Hands Spark Investigation in Germany. https://edition.cnn.com/2019/09/16/health/hand-deformities-babies-gelsenkirchen-germany-intl-scli-grm/index.html.

[83] VACTERL Association. https://www.gosh.nhs.uk/conditions-and-treatments/conditions-we-treat/vacterl-association-0/.

[84] Reece A.S., Hulse G.K. (2022). Epidemiological Patterns of Cannabis- and Substance- Related General Congenital Anomalies Across Europe 2010–2019: Space-Time and Causal Inference Study.

[85] Eurocat Data: Prevalence Charts and Tables. https://eu-rd-platform.jrc.ec.europa.eu/eurocat/eurocat-data/prevalence_en.

[86] Global Health Observatory. https://www.who.int/data/gho/data/indicators/indicator-details/GHO/total-(recorded-unrecorded)-alcohol-per-capita-(15-)-consumption.

[87] European Monitoring Centre for Drugs and Drug Addiction (EMCDDA) Statistical Bulletin 2021—Prevalence of Drug Use. https://www.emcdda.europa.eu/data/stats2021/gps_en.

[88] The World Bank Crude Data: Adjusted Net National Income per Capita (Current US$). https://data.worldbank.org/indicator/NY.ADJ.NNTY.PC.CD.

[89] R: A Language and Environment for Statistical Computing. https://cran.r-project.org/.

[90] Wickham H., Averick M., Bryan J., Chang W., McGowan L.D., Francios R., Groelmund G., Hayes A., Henry L., Hester J. (2019). Welcome to the Tidyverse. J. Open Source Softw..

[91] Pebesma E. (2018). Simple Features for R: Standardized Support for Spatial Vector Data. R J..

[92] Viridis: Default Color Maps from ‘matplotlib’. https://CRAN.R-project.org/package=viridis.

[93] Colorplaner: Ggplot2 Extension to Visualize Two Variables Per Color Aesthetic Through Colorspace Projection. https://github.com/wmurphyrd/colorplaner.

[94] Pinheiro J., Bates D., DebRoy S., Sarkar D., Team R.C. (2020). Linear and Nonlinear Mixed Effects Models. R Package Version.

[95] Broom.Mixed: Tidying Methods for Mixed Models. http://github.com/bbolker/broom.mixed.

[96] Broom: Convert Statistical Objects into Tidy Tibbles. https://CRAN.R-project.org/package=broom.

[97] Leeper T.J., Leeper T.J. (2021). Margins: Marginal Effects for Model Objects.

[98] Wright M.N., Ziegler A. (2017). ranger: A Fast Implementation of Random Forests for High Dimensional Data in C++ and R. J. Stat. Softw..

[99] Greenwell B.M., Boehmke B.C. (2021). Variable Importance Plots—An Introduction to the vip Package. R J..

[100] Package ‘plm’. https://cran.r-project.org/web/packages/plm/plm.pdf.

[101] Bivand R., Anselin L., Berke O., Bernat A., Carvalho M., Chun Y., Dormann C., Dray S., Halbersma R., Lewis-Koh N. (2007). The spdep Package.

[102] Millo G., Piras G. (2012). splm: Spatial Panel Data Models in R. J. Stat. Softw..

[103] Millo G., Piras G. (2018). Package ‘splm’.

[104] Croissant Y., Millo G. (2019). Panel Data Econometrics with R.

[105] Wal W., Geskus R. (2011). ipw: An R Package for Inverse Probability Weighting. J. Stat. Softw..

[106] VanderWeele T.J., Ding P. (2017). Sensitivity Analysis in Observational Research: Introducing the E-Value. Ann. Intern. Med..

[107] VanderWeele T.J., Martin J.N., Mathur M.B. (2020). **E**-values and incidence density sampling. Epidemiology.

[108] VanderWeele T.J., Mathur M.B. (2020). Commentary: Developing best-practice guidelines for the reporting of E-values. Int. J. Epidemiol..

[109] VanderWeele T.J., Ding P., Mathur M. (2019). Technical Considerations in the Use of the E-Value. J. Causal Inference.

[110] Pearl J., Mackaenzie D. (2019). The Book of Why. The New Science of Cause and Effect.

[111] Package ‘EValue’. https://cran.r-project.org/web/packages/EValue/EValue.pdf.

[112] Hill A.B. (1965). The Environment and Disease: Association or Causation?. Proc. R Soc. Med..

[113] Reece A.S., Hulse G.K. (2019). Effect of Cannabis Legalization on US Autism Incidence and Medium Term Projections. Clin. Pediatrics Open Access.

[114] Reece A.S. (2018). Rapid Response: Known Cannabis Teratogenicity Needs to be Carefully Considered. BMJ.

[115] Reece A.S., Hulse G.K. (2019). Impacts of cannabinoid epigenetics on human development: Reflections on Murphy et al. ‘cannabinoid exposure and altered DNA methylation in rat and human sperm’ epigenetics. Epigenetics.

[116] Fish E.W., Murdaugh L.B., Zhang C., Boschen K.E., Boa-Amponsem O., Mendoza-Romero H.N., Tarpley M., Chdid L., Mukhopadhyay S., Cole G.J. (2019). Cannabinoids exacerbate alcohol teratogenesis by a CB1-hedgehog interaction. Sci. Rep..

[117] Aguado T., Romero E., Monory K., Palazuelos J., Sendtner M., Marsicano G., Lutz B., Guzmán M., Galve-Roperh I. (2007). The CB1 cannabinoid receptor mediates excitotoxicity-induced neural progenitor proliferation and neurogenesis. J. Biol. Chem..

[118] Williams E.J., Walsh F.S., Doherty P. (2003). The FGF receptor uses the endocannabinoid signaling system to couple to an axonal growth response. J. Cell Biol..

[119] Birerdinc A., Jarrar M., Stotish T., Randhawa M., Baranova A. (2013). Manipulating molecular switches in brown adipocytes and their precursors: A therapeutic potential. Prog. Lipid Res..

[120] Richard D., Picard F. (2011). Brown fat biology and thermogenesis. Front. Biosci..

[121] Xu T.R., Yang Y., Ward R., Gao L., Liu Y. (2013). Orexin receptors: Multi-functional therapeutic targets for sleeping disorders, eating disorders, drug addiction, cancers and other physiological disorders. Cell. Signal..

[122] Fraher D., Ellis M.K., Morrison S., McGee S.L., Ward A.C., Walder K., Gibert Y. (2015). Lipid Abundance in Zebrafish Embryos Is Regulated by Complementary Actions of the Endocannabinoid System and Retinoic Acid Pathway. Endocrinology.

[123] Kučukalić S., Ferić Bojić E., Babić R., Avdibegović E., Babić D., Agani F., Jakovljević M., Kučukalić A., Bravo Mehmedbašić A., Šabić Džananović E. (2019). Genetic susceptibility to posttraumatic stress disorder: Analyses of the oxytocin receptor, retinoic acid receptor-related orphan receptor A and cannabinoid receptor 1 genes. Psychiatr. Danub..

[124] Lee Y.S., Jeong W.I. (2012). Retinoic acids and hepatic stellate cells in liver disease. J. Gastroenterol. Hepatol..

[125] Ungricht R., Guibbal L., Lasbennes M.C., Orsini V., Beibel M., Waldt A., Cuttat R., Carbone W., Basler A., Roma G. (2022). Genome-wide screening in human kidney organoids identifies developmental and disease-related aspects of nephrogenesis. Cell Stem Cell.

[126] Carlson B.M. (2019). Human Embryology and Developmental Biology.

