# Supplementary material for: Epidemiological Patterns of Cannabis- and Substance- Related Congenital Uronephrological Anomalies in Europe: Geospatiotemporal and Causal Inferential Study
Source: Int J Environ Res Public Health. 2022 Oct 23;19(21):13769. doi: 10.3390/ijerph192113769 (PMC9657099; doi:10.3390/ijerph192113769)
Supplement: Supplementary file 1 [file ijerph-19-13769-s001.zip › ijerph-1951474-supplementary.pdf]

Supplementary Material for:

Epidemiological Patterns of Cannabis- and Substance- Related Congenital Uronephrological Anomalies in Europe: Geospatiotemporal and Causal Inferential Study

Albert Stuart Reece and Gary Kenneth Hulse

University of Western Australia and
Edith Cowan University, Western Australia

Supplementary Tables

Contents

Supplementary Table Number	Contents
S1	Overall Study Profile
S2	Daily Cannabis Use - Raw Data
S3	Daily Cannabis Use - Interpolated Data
S4	All regression slopes and results from bivariate analyses
S5	Variable Importance Tables from Ranger random forest regression - Uronephrological anomalies
S6	Variable Importance Tables from Ranger random forest regression - Multicystic renal disease
S7	Variable Importance Tables from Ranger random forest regression - Bilateral renal agenesis
S8	Variable Importance Tables from Ranger random forest regression - Hydronephrosis
S9	Variable Importance Tables from Ranger random forest regression - Congenital posterior urethral valve
S10	Inverse probability weighted panel regression results - Uronephrological anomalies
S11	Inverse probability weighted panel regression results - Multicystic renal disease
S12	Inverse probability weighted panel regression results - Bilateral renal agenesis
S13	Inverse probability weighted panel regression results - Hydronephrosis
S14	Inverse probability weighted panel regression results - Congenital posterior urethral valve

Supplementary Table S1.: Overall Variables and Metrics

Variable	Value
Sample Size	839
Country (%)	
Belgium	70 (8.3)
Bulgaria	70 (8.3)
Croatia	56 (6.7)
Finland	30 (3.6)
France	70 (8.3)
Germany	70 (8.3)
Hungary	18 (2.1)
Italy	70 (8.3)
Netherlands	63 (7.5)
Norway	70 (8.3)
Poland	70 (8.3)
Portugal	70 (8.3)
Spain	70 (8.3)
Sweden	42 (5.0)
Year (Range)	2010: 2019
Anomaly (%)	
Bilateral renal agenesis including Potter syndrome	122 (14.5)
Bladder exstrophy and/or epispadia	122 (14.5)
Congenital hydronephrosis	122 (14.5)
Hypospadias	107 (12.8)
Multicystic renal dysplasia	122 (14.5)
Posterior urethral valve and/or prune belly	122 (14.5)
Urinary	122 (14.5)
Anomaly_Rate (mean (SD))	10.61 (14.37)
Tobacco (mean (SD))	22.79 (5.46)
Alcohol (p.c.L/Yr) (mean (SD))	10.77 (1.83)
Amphetamines (% Use) (median [IQR])	0.60 [0.29, 0.80]
Cocaine (% Use) (median [IQR])	0.72 [0.40, 1.20]
Last_Month_Cannabis (% Use) (mean (SD))	0.04 (0.02)
Cannabis_Herb_THC_Content (%) (mean (SD))	0.09 (0.03)
Cannabis_Resin_THC_Content (%) (mean (SD))	0.16 (0.09)
%_Daily_Cannabis_ Use (mean (SD))	0.01 (0.01)
%_Daily_Cannabis_Use_Interpolated (mean (SD))	0.01 (0.01)
Last_Month_Cannabis_x_Herb_THC_Content (mean (SD))	0.04 (0.03)
Last_Month_Cannabis_x_Resin_THC_Content (mean (SD))	0.13 (0.12)
Last_Month_Cannabis_x_Herb_THC_Content_x_Daily_Use_Interpolated (median [IQR])	0.02 [0.00, 0.06]

Last_Month_Cannabis_x_Resin_THC_Content_x_Daily_Use_Interpolated (median [IQR])	0.03 [0.01, 0.13]
Trend_in_Daily_Cannabis_Use = Increasing (%)	609 (72.6)
Mean_Annual_Household_Income (mean (SD))	30163.14 (17332.69)

Supplementary Table S2.: Data on Daily Cannabis Use

Country	2010	2011	2012	2013	2014	2015	2016	2017	2018	2019
Belgium	-	-	-	0.0054	-	-	-	-	0.0132	-
Bulgaria	-	-	0.0020	-	-	-	0.0016	-	-	-
Croatia	-	-	0.0050	-	-	0.0078	-	-	-	-
Finland	0.0020	-	-	-	0.0031	-	-	-	-	-
France	0.0150	-	-	-	0.0217	-	-	0.0199	-	-
Germany	-	-	0.0060	-	-	0.0067	-	-	0.0084	-
Hungary	-	-	-	-	-	-	-	-	-	-
Italy	-	-	0.0030	-	0.0094	-	-	0.0108	-	-
Netherlands	-	0.0146	0.0146	0.0146	0.0146	0.0146	0.0133	0.0126	0.0190	0.0200
Norway	0.0030	0.0030	0.0030	0.0030	0.0037	0.0044	0.0053	0.0016	0.0068	0.0038
Poland	0.0040	-	0.0010	-	0.0018	-	-	-	-	-
Portugal	-	-	0.0050	-	-	-	0.0002	-	-	-
Spain	-	0.0250	-	0.0260	-	0.0285	-	0.0372	-	-
Sweden	-	-	-	-	-	-	-	-	-	-

Supplementary Table S3.: Interpolated Data on Daily Cannabis Use

Country	2010	2011	2012	2013	2014	2015	2016	2017	2018	2019
Belgium	0.0054	0.0054	0.0054	0.0054	0.0070	0.0085	0.0101	0.0116	0.0132	0.0132
Bulgaria	0.0020	0.0020	0.0020	0.0019	0.0018	0.0017	0.0016	0.0016	0.0016	0.0016
Croatia	0.0050	0.0050	0.0050	0.0059	0.0069	0.0078	0.0092	0.0107	-	-
Finland	0.0020	0.0023	0.0026	0.0028	0.0031	-	-	-	-	-
France	0.0150	0.0167	0.0184	0.0200	0.0217	0.0211	0.0205	0.0199	0.0199	0.0199
Germany	0.0060	0.0060	0.0060	0.0062	0.0065	0.0067	0.0073	0.0078	0.0084	0.0084
Hungary	0.0007	0.0007	0.0007	-	-	-	-	-	-	-
Italy	0.0030	0.0030	0.0030	0.0062	0.0094	0.0099	0.0103	0.0108	0.0108	0.0108
Netherlands	-	0.0146	0.0146	0.0146	0.0146	0.0146	0.0133	0.0126	0.0190	0.0200
Norway	0.0030	0.0030	0.0030	0.0030	0.0037	0.0044	0.0053	0.0016	0.0068	0.0038
Poland	0.0040	0.0025	0.0010	0.0014	0.0018	0.0018	0.0018	0.0018	0.0018	0.0018
Portugal	0.0050	0.0050	0.0050	0.0038	0.0026	0.0014	0.0002	0.0002	0.0002	0.0002
Spain	0.0250	0.0250	0.0255	0.0260	0.0273	0.0285	0.0329	0.0372	0.0372	0.0372
Sweden	-	-	-	-	-	-	-	-	-	-

Supplementary Table S4.: Regression Results and E-Values for Substances and Cannabinoids

Anomaly	Substance	Mean Anomaly Rate	Estimate	Std.Error	Sigma	t_statistic	P_Value	E-Value Estimate	E-Value Lower Bound
Multicystic Renal Dys	Daily.Interpol.	3.4655	30.4447	9.5953	0.9007	3.1729	0.0019	4.57E+13	2.66E+05
Hydronephrosis	Daily.Interpol.	13.8455	27.8771	8.7897	0.8250	3.1716	0.0020	4.52E+13	2.62E+05
Urinary	Daily.Interpol.	35.4475	14.3684	5.3583	0.5029	2.6815	0.0084	3.90E+11	2.27E+03
Multicystic Renal Dys	LMCannabis_Herb	3.4655	11.4522	3.0267	0.8688	3.7837	2.43E-04	3.24E+05	656.07
Hypospadias	Herb	19.2037	4.9196	1.3727	0.4546	3.5838	5.15E-04	3.78E+04	174.71
Multicystic Renal Dys	LM_Cannabis	3.4655	11.4477	3.6514	0.8837	3.1352	0.0022	2.63E+05	167.94
Urinary	LMCannabis_Herb	35.4475	5.0871	1.7232	0.4947	2.9521	0.0038	2.32E+04	46.53
Hydronephrosis	Herb	13.8455	6.5820	2.1094	0.8202	3.1204	0.0023	2.97E+03	30.00
Urinary	Herb	35.4475	3.8164	1.2706	0.4941	3.0036	0.0032	2.26E+03	22.70
Hydronephrosis	LMCannabis_Herb	13.8455	7.3128	2.8950	0.8310	2.5260	0.0128	6.01E+03	11.66
Multicystic Renal Dys	LMCannabis_Resin	3.4655	3.0558	0.7366	0.8820	4.1488	6.81E-05	46.30	10.06
Multicystic Renal Dys	Herb	3.4655	5.5861	2.3083	0.8976	2.4200	0.0170	575.83	5.37
Bilat Renal Agenesis	Herb	1.3617	3.9363	1.6424	0.6386	2.3967	0.0181	545.31	5.05
Multicystic Renal Dys	LM.Cannabis_x_Herb.THC_x_Daily.Interpol.	3.4655	2.8187	1.0685	0.9122	2.6379	0.0095	32.77	3.56
Posterior Ureth Valv	Herb	1.3062	3.8400	1.7052	0.6630	2.2519	0.0261	388.43	3.41
Multicystic Renal Dys	LM.Cannabis_x_Resin.THC_x_Daily.Interpol.	3.4655	1.6437	0.4955	0.9272	3.3171	0.0013	9.51	3.29
Hydronephrosis	LM.Cannabis_x_Herb.THC_x_Daily.Interpol.	13.8455	2.4167	0.9825	0.8388	2.4597	0.0154	27.01	2.81
Urinary	LMCannabis_Resin	35.4475	1.0733	0.4108	0.4919	2.6129	0.0103	14.05	2.68

Hydronephrosis	Cocaine	13.8455	0.4916	0.0962	0.7729	5.1079	1.24E-06	2.97	2.21
Urinary	LM.Cannabis_x_Herb.THC_x_Daily.Interpol.	35.4475	1.3384	0.5944	0.5074	2.2517	0.0263	21.54	2.08
Urinary	Cocaine	35.4475	0.2657	0.0590	0.4738	4.5041	1.56E-05	2.72	2.00
Urinary	LM.Cannabis_x_Resin.THC_x_Daily.Interpol.	35.4475	0.6738	0.2664	0.4985	2.5291	0.0130	6.30	1.97
Multicystic Renal Dys	Cocaine	3.4655	0.4660	0.1063	0.8533	4.3857	2.50E-05	2.67	1.96
Hydronephrosis	LM.Cannabis_x_Resin.THC_x_Daily.Interpol.	13.8455	1.1656	0.4627	0.8659	2.5189	0.0134	6.26	1.96
Hypospadias	Cocaine	19.2037	0.2230	0.0608	0.4534	3.6679	3.86E-04	2.50	1.77
Posterior Ureth Valv	LMCannabis_Resin	1.3062	1.2729	0.5745	0.6880	2.2154	0.0289	10.24	1.73
Hydronephrosis	LMCannabis_Resin	13.8455	1.5445	0.7215	0.8639	2.1407	0.0346	9.65	1.57
Blader Extr / Epispad	LMCannabis_Resin	0.6657	0.8171	0.3848	0.4608	2.1235	0.0361	9.52	1.53
Hydronephrosis	Resin	13.8455	1.9876	0.9624	0.8652	2.0652	0.0414	15.66	1.48
Urinary	Log(Amphetamine)	35.4475	0.1765	0.0622	0.4959	2.8377	0.0053	2.11	1.45
Blader Extr / Epispad	Cocaine	0.6657	0.1511	0.0548	0.4405	2.7545	0.0068	2.07	1.42
Hydronephrosis	Log(Amphetamine)	13.8455	0.2789	0.1039	0.8283	2.6856	0.0083	2.06	1.39
Hypospadias	Log(Amphetamine)	19.2037	0.1577	0.0603	0.4667	2.6142	0.0103	2.06	1.38
Multicystic Renal Dys	Resin	3.4655	2.1084	1.0383	0.9334	2.0306	0.0448	15.11	1.37
Bilat Renal Agenesis	Cocaine	1.3617	0.2017	0.0793	0.6368	2.5443	0.0122	2.00	1.34
Posterior Ureth Valv	Annual_Alcohol	1.3062	0.0958	0.0325	0.6537	2.9450	0.0039	1.55	1.26
Multicystic Renal Dys	Log(Amphetamine)	3.4655	0.2537	0.1129	0.9005	2.2464	0.0265	1.91	1.22
Multicystic Renal Dys	Annual_Alcohol	3.4655	0.0886	0.0450	0.9047	1.9686	0.0513	1.41	1.02
Posterior Ureth Valv	Tobacco	1.3062	0.0035	0.0113	0.6766	0.3136	0.7544	1.07	1
Hypospadias	Tobacco	19.2037	0.0044	0.0083	0.4810	0.5230	0.6021	1.10	1
Multicystic Renal Dys	Tobacco	3.4655	0.0141	0.0152	0.9159	0.9266	0.3560	1.13	1
Hypospadias	LM.Cannabis_x_Resin.THC_x_Daily.Interpol.	19.2037	0.0089	0.2638	0.4911	0.0336	0.9733	1.15	1
Bilat Renal Agenesis	Annual_Alcohol	1.3617	0.0241	0.0324	0.6522	0.7436	0.4586	1.22	1
Urinary	Annual_Alcohol	35.4475	0.0268	0.0254	0.5099	1.0558	0.2932	1.28	1

Posterior Ureth Valv	Log(Amphetamine)	1.3062	0.1037	0.0844	0.6727	1.2291	0.2214	1.57	1
Posterior Ureth Valv	LM.Cannabis_x_Herb.THC: LM.Cannabis_x_Resin.THC_x_Daily.Interpol.	1.3062	0.1507	0.0832	0.6678	1.8118	0.0725	1.76	1
Bilat Renal Agenesis	Log(Amphetamine)	1.3617	0.1512	0.0808	0.6444	1.8708	0.0638	1.78	1
Blader Extr / Epispad	Log(Amphetamine)	0.6657	0.1086	0.0561	0.4473	1.9371	0.0551	1.80	1
Hypospadias	LMCannabis_Resin	19.2037	0.2717	0.4288	0.4901	0.6337	0.5278	2.70	1
Bilat Renal Agenesis	LM.Cannabis_x_Resin.THC_x_Daily.Interpol.	1.3617	0.3876	0.3645	0.6820	1.0635	0.2902	2.74	1
Hypospadias	LM_Cannabis	19.2037	0.4004	2.2438	0.4815	0.1785	0.8587	3.68	1
Posterior Ureth Valv	LM.Cannabis_x_Resin.THC_x_Daily.Interpol.	1.3062	0.6228	0.3798	0.7108	1.6396	0.1043	3.86	1
Posterior Ureth Valv	LM.Cannabis_x_Herb.THC_x_Daily.Interpol.	1.3062	0.6219	0.8068	0.6888	0.7708	0.4424	3.98	1
Blader Extr / Epispad	LM.Cannabis_x_Resin.THC_x_Daily.Interpol.	0.6657	0.4929	0.2541	0.4755	1.9397	0.0553	4.58	1
Bilat Renal Agenesis	Daily.Interpol.	1.3617	0.7176	0.5518	0.6607	1.3004	0.1963	4.82	1
Bilat Renal Agenesis	LM.Cannabis_x_Herb.THC_x_Daily.Interpol.	1.3617	0.7671	0.7832	0.6686	0.9795	0.3294	5.13	1
Blader Extr / Epispad	LM.Cannabis_x_Herb.THC_x_Daily.Interpol.	0.6657	0.7704	0.5409	0.4618	1.4243	0.1571	8.60	1
Bilat Renal Agenesis	LM_Cannabis	1.3617	1.0978	2.6992	0.6533	0.4067	0.6849	8.70	1
Posterior Ureth Valv	Resin	1.3062	1.2371	0.7736	0.6954	1.5991	0.1128	9.57	1
Hypospadias	Resin	19.2037	0.8786	0.5633	0.4850	1.5597	0.1222	9.87	1
Blader Extr / Epispad	Resin	0.6657	0.8604	0.5167	0.4645	1.6652	0.0988	10.27	1
Urinary	Resin	35.4475	1.0041	0.5561	0.4999	1.8057	0.0738	11.92	1
Bilat Renal Agenesis	Resin	1.3617	1.3272	0.7295	0.6558	1.8193	0.0717	12.09	1
Blader Extr / Epispad	Herb	0.6657	1.2940	1.1621	0.4519	1.1135	0.2677	26.58	1
Posterior Ureth Valv	Resin	1.3062	2.0478	2.7906	0.6754	0.7338	0.4645	31.07	1
Blader Extr / Epispad	LM_Cannabis	0.6657	2.0609	1.8672	0.4519	1.1037	0.2719	126.36	1
Hypospadias	Daily.Interpol.	19.2037	2.3279	5.2605	0.4812	0.4425	0.6590	162.83	1
Bilat Renal Agenesis	LMCannabis_Herb	1.3617	3.2735	2.2577	0.6481	1.4500	0.1497	197.80	1

Urinary	LM_Cannabis	35.4475	2.6317	2.1031	0.5090	1.2514	0.2132	220.51	1
Hypospadias	LMCannabis_Herb	19.2037	2.6928	1.8415	0.4768	1.4623	0.1467	340.78	1
Posterior Ureth Valv	LMCannabis_Herb	1.3062	3.8311	2.3321	0.6694	1.6428	0.1030	364.92	1
Blader Extr / Epispad	LMCannabis_Herb	0.6657	2.6306	1.5639	0.4489	1.6820	0.0952	413.34	1
Hydronephrosis	LM_Cannabis	13.8455	6.2040	3.4779	0.8417	1.7838	0.0770	1.64E+03	1
Posterior Ureth Valv	Daily.Interpol.	1.3062	8.1738	7.3175	0.6869	1.1170	0.2664	1.01E+05	1
Bilat Renal Agenesis	Daily.Interpol.	1.3617	8.1612	7.1125	0.6676	1.1475	0.2536	1.36E+05	1
Blader Extr / Epispad	Daily.Interpol.	0.6657	9.4994	4.8825	0.4583	1.9456	0.0542	3.11E+08	1
Urinary	Tobacco	35.4475	-0.0064	0.0085	0.5111	-0.7516	0.4538	1.12	-
Hypospadias	LM.Cannabis_x_Herb.THC_x_Daily.Interpol.	19.2037	-0.0102	0.5719	0.4816	-0.0179	0.9858	1.16	-
Bilat Renal Agenesis	Tobacco	1.3617	-0.0140	0.0108	0.6492	-1.2982	0.1967	1.16	-
Blader Extr / Epispad	Tobacco	0.6657	-0.0099	0.0075	0.4509	-1.3255	0.1875	1.16	-
Blader Extr / Epispad	Annual_Alcohol	0.6657	-0.0163	0.0225	0.4532	-0.7233	0.4709	1.22	-
Hydronephrosis	Annual_Alcohol	13.8455	-0.0602	0.0421	0.8456	-1.4314	0.1549	1.33	-
Hydronephrosis	Tobacco	13.8455	-0.0571	0.0132	0.7932	-4.3274	3.14E-05	1.34	-
Hypospadias	Annual_Alcohol	19.2037	-0.0866	0.0233	0.4528	-3.7122	3.31E-04	1.67	-

Table Notes: LM – Last Month

Supplementary Table S5.: Random Forest Regression Table for Congenital Uronephrological Anomalies

Variable	Importance
Income	5.3569
LM.Cannabis_x_Herb.THC_x_Daily.Interpol.	3.0641
LM.Cannabis_x_Resin.THC_x_Daily.Interpol.	3.0386
Alcohol	2.8534
Daily.Interpol.	2.8279
LM.Cannabis_x_Herb.THC	2.0332
Cocaine	2.0183
Tobacco	1.9990
LM.Cannabis_x_Resin.THC	1.6984
Amphetamines	1.6354
Herb	1.5265
LM.Cannabis	1.4531
Resin	1.1507

Table Notes: L.M. – Last Month

Supplementary Table S6.: Random Forest Regression Table for Multicystic Renal Disease

Variable	Importance
Income	11.0184
LM.Cannabis_x_Herb.THC_x_Daily.Interpol.	10.0873
Alcohol	9.9802
LM.Cannabis_x_Resin.THC_x_Daily.Interpol.	9.4842
Daily.Interpol.	9.2637
LM.Cannabis_x_Resin.THC	7.1126
Resin	6.2535
Cocaine	6.2476
LM.Cannabis	5.7416
LM.Cannabis_x_Herb.THC	5.7316
Amphetamines	5.6027
Tobacco	5.5267
Herb	4.7051

Table Notes: L.M. – Last Month

Supplementary Table S7.: Random Forest Regression Table for Bilateral Renal Agenesis

**Variable**	**Importance**
Income	7.6470
Tobacco	5.5964
LM.Cannabis_x_Resin.THC_x_Daily.Interpol.	5.0778
LM.Cannabis_x_Resin.THC	3.6514
Herb	3.6059
Resin	3.4798
LM.Cannabis_x_Herb.THC_x_Daily.Interpol.	3.0782
Alcohol	2.9611
Daily.Interpol.	2.6024
LM.Cannabis	2.4464
Amphetamines	2.4454
Cocaine	2.1307
LM.Cannabis_x_Herb.THC	2.1096

Table Notes: L.M. – Last Month

Supplementary Table S8.: Random Forest Regression Table for Hydronephrosis

Variable	Importance
Income	17.4719
Tobacco	14.8985
Daily.Interpol.	7.8667
Cocaine	6.1065
Amphetamines	5.6626
LM.Cannabis_x_Resin.THC_x_Daily.Interpol.	5.3265
Resin	4.1168
LM.Cannabis_x_Herb.THC_x_Daily.Interpol.	3.9855
Alcohol	3.3670
Herb	3.3324
LM.Cannabis_x_Resin.THC	3.0574
LM.Cannabis_x_Herb.THC:<br>LM.Cannabis_x_Resin.THC_x_Daily.Interpol.	2.9906
LM.Cannabis	2.1353

Table Notes: L.M. – Last Month

Supplementary Table S9.: Random Forest Regression Table for Congenital Posterior Urethral Valve

Variable	Importance
Income	9.9239
Resin	5.8367
Alcohol	4.6922
LM.Cannabis_x_Resin.THC_x_Daily.Interpol.	4.0142
Resin	3.4977
Daily.Interpol.	3.4056
Herb	3.2753
LM.Cannabis_x_Resin.THC	3.1680
LM.Cannabis_x_Herb.THC_x_Daily.Interpol.	3.1308
LM.Cannabis_x_Herb.THC	2.5791
Amphetamines	2.5485
LM.Cannabis	2.4869
Cocaine	2.3320

Table Notes: L.M. – Last Month

Supplementary Table S10.: Panel Regression Results for Congenital Uronephrological Anomalies

Parameters			Model Parameters	
**Term**	**Estimate (C.I.)**	**P-Value**	**Parameter**	**Value**
**Additive**				
**LAnomRt ~ Tob + Alc + LpmHerbDailyInt + LpmResinDailyInt + pmHerb + pm.Daily.Intpltd + LAmph + LCocc + MHY**				
LM.Cannabis_x_Herb.THC_x_Daily.Interpol.	0.77 (0.32, 1.23)	0.0012	Adj.R.Squared	0.1605
LM.Cannabis_x_Resin.THC_x_Daily.Interpol.	-0.88 (-1.4, -0.36)	0.0013	Statistic	7.7943
LM.Cannabis_x_Herb.THC	9.69 (5.44, 13.94)	1.91E-05	Deg.Freedom	6,115
Amphetamines	0.19 (0.07, 0.31)	0.0031	P-Value	4.76E-07
Cocaine	-0.13 (-0.23, -0.03)	0.0120		
Income	0 (0, 0)	1.16E-05		
**Interactive**				
**Rate ~ Tobacco * LM.Cannabis_x_Herb.THC_x_Daily.Interpol. * LM.Cannabis_x_Resin.THC_x_Daily.Interpol. + LM.Cannabis_x_Resin.THC + Alcohol + Daily.Interpol. + Amphetamines + Cocaine + Income**				
LM.Cannabis_x_Herb.THC_x_Daily.Interpol.	1.09 (0.21, 1.97)	0.0165	Adj.R.Squared	0.1137
LM.Cannabis_x_Resin.THC_x_Daily.Interpol.	-2.59 (-4.26, -0.92)	0.0029	Statistic	6.3184
LM.Cannabis_x_Resin.THC	1.82 (0.5, 3.14)	0.0079	Deg.Freedom	5,116
Income	0 (0, 0)	0.0014	P-Value	3.19E-05
Tobacco: LM.Cannabis_x_Resin.THC_x_Daily.Interpol.	0.06 (0.02, 0.11)	0.0092		
**2 Lags**				
**Rate ~ Tobacco * LM.Cannabis_x_Resin.THC_x_Daily.Interpol. * Resin + LM.Cannabis_x_Herb.THC_x_Daily.Interpol. + Alcohol + Daily.Interpol. + Amphetamines + Cocaine + Income**				
Resin	-5.6 (-8.56, -2.64)	0.0004	Adj.R.Squared	0.4051

Income	0 (0, 0)	0.0002	Statistic	11.2479
Tobacco: Resin	0.26 (0.14, 0.38)	6.32E-05	Deg.Freedom	3,90
			P-Value	2.45E-06

Table Notes:
L.M. – Last Month
pm – Past Month
LpmHerbDailyInt – Log (Last Month Cannabis x Herb THC x Daily Cannabis Use Interpolated)
LpmResinDailyInt – Log (Last Month Cannabis x Resin THC x Daily Cannabis Use Interpolated)
L - Log

Supplementary Table S11.: Panel Regression Results for Multicystic Renal Disease

Parameters			Model Parameters	
**Term**	**Estimate (C.I.)**	**P-Value**	**Parameter**	**Value**
**Additive**				
**Rate ~ Tobacco + Alcohol + LM.Cannabis_x_Herb.THC_x_Daily.Interpol. + LM.Cannabis_x_Resin.THC_x_Daily.Interpol. + LM.Cannabis_x_Resin.THC + Daily.Interpol. + Amphetamines + Cocaine + Income**				
Tobacco	0.05 (0.02, 0.07)	0.0002	Adj.R.Squared	0.1221
LM.Cannabis_x_Resin.THC	2.03 (1.37, 2.7)	2.61E-08	Statistic	10.9555
Daily.Interpol.	-41.06 (-60.43, -21.69)	6.21E-05	Deg.Freedom	4,117
Cocaine	0.56 (0.27, 0.84)	0.0002	P-Value	1.41E-07
**Interactive**				
**Rate ~ Tobacco * Daily.Interpol. * LM.Cannabis_x_Resin.THC_x_Daily.Interpol. + LM.Cannabis_x_Herb.THC_x_Daily.Interpol. + LM.Cannabis_x_Resin.THC + Alcohol + Amphetamines + Cocaine + Income**				
Tobacco	0.1 (0.06, 0.13)	2.93E-07	Adj.R.Squared	0.05979
Daily.Interpol.	121.57 (1.39, 241.74)	0.0498	Statistic	8.9172
Cocaine	0.87 (0.51, 1.22)	6.21E-06	Deg.Freedom	6,115
Tobacco: Daily.Interpol.	-7.57 (-12.16, -2.99)	0.0016	P-Value	5.47E-08
Daily.Interpol.: LM.Cannabis_x_Resin.THC_x_Daily.Interpol.	-199.35 (-350.53, -48.17)	0.011		
Tobacco: Daily.Interpol.: LM.Cannabis_x_Resin.THC_x_Daily.Interpol.	9.4 (2.77, 16.02)	0.0063		
**2 Lags**				
**Rate ~ Tobacco * Daily.Interpol. + LM.Cannabis_x_Resin.THC_x_Daily.Interpol. + LM.Cannabis_x_Resin.THC + LM.Cannabis_x_Herb.THC_x_Daily.Interpol. + Alcohol + Amphetamines + Cocaine + Income**				
Tobacco	0.13 (0.06, 0.19)	0.0001	Adj.R.Squared	0.1432
Daily.Interpol.	187 (8.05, 365.95)	0.0433	Statistic	6.8338

LM.Cannabis_x_Resin.THC_x_Daily.Interpol.	-3.04 (-5.74, -0.34)	0.0300	Deg.Freedom	7,86
LM.Cannabis_x_Resin.THC	4.52 (1.17, 7.87)	0.0097	P-Value	1.88E-06
LM.Cannabis_x_Herb.THC_x_Daily.Interpol.	2.7 (0.35, 5.05)	0.0269		
Income	0 (0, 0)	0.0042		
Tobacco: Daily.Interpol.	-7.67 (-14.67, -0.67)	0.0343		

Table Notes: L.M. – Last Month

Supplementary Table S12.: Panel Regression Results for Bilateral Renal Agenesis

Parameters			Model Parameters	
**Term**	**Estimate (C.I.)**	**P-Value**	**Parameter**	**Value**
**Additive**				
**Rate ~ Tobacco + Alcohol + LM.Cannabis_x_Herb.THC_x_Daily.Interpol. + LM.Cannabis_x_Resin.THC_x_Daily.Interpol. + LM.Cannabis_x_Resin.THC + Resin + Daily.Interpol. + Herb + Amphetamines + Cocaine + Income**				
Resin	1 (0.12, 1.87)	0.0273	Adj.R.Squared	0.0047
Daily.Interpol.	30.4 (14.47, 46.33)	0.0003	Statistic	20.1265
Cocaine	-0.45 (-0.76, -0.15)	0.0042	Deg.Freedom	4,117
Income	0 (0, 0)	3.95E-05	P-Value	1.24E-12
**Interactive**				
**Rate ~ Tobacco * Daily.Interpol. + LM.Cannabis_x_Herb.THC_x_Daily.Interpol. + LM.Cannabis_x_Resin.THC + LM.Cannabis_x_Resin.THC_x_Daily.Interpol. + Alcohol + Herb + Amphetamines + Cocaine + Income**				
Tobacco	0.05 (0.01, 0.1)	0.0254	Adj.R.Squared	0.0465
Daily.Interpol.	207 (137.22, 276.78)	5.10E-08	Statistic	21.7433
Cocaine	-0.35 (-0.61, -0.09)	0.0101	Deg.Freedom	5,116
Income	0 (0, 0)	0.0014	P-Value	2.62E-15
Tobacco: Daily.Interpol.	-7.55 (-10.69, -4.41)	6.95E-06		
**2 Lags**				
**Rate ~ Tobacco * Daily.Interpol. + LM.Cannabis_x_Resin.THC_x_Daily.Interpol. + LM.Cannabis_x_Herb.THC_x_Daily.Interpol. + LM.Cannabis_x_Resin.THC + Alcohol + Amphetamines + Cocaine + Income**				
LM.Cannabis_x_Resin.THC	2.41 (1.91, 2.91)	4.60E-15	Adj.R.Squared	0.1301
Cocaine	0.47 (0.2, 0.74)	0.0009	Statistic	30.9486

Tobacco: Daily.Interpol.	-1.31 (-1.93, -0.69)	7.72E-05	Deg.Freedom	3,90
			P-Value	7.71E-14

Table Notes: L.M. – Last Month

Supplementary Table S13.: Panel Regression Results for Congenital Hydronephrosis

**Parameters**			**Model Parameters**	
**Term**	**Estimate (C.I.)**	**P-Value**	**Parameter**	**Value**
**Additive**				
**Rate ~ Tobacco + Alcohol + LM.Cannabis_x_Herb.THC_x_Daily.Interpol. + LM.Cannabis_x_Resin.THC_x_Daily.Interpol. + LM.Cannabis_x_Resin.THC + Daily.Interpol. + Resin + Herb + Amphetamines + Cocaine + Income**				
Daily.Interpol.	53 (31.24, 74.76)	5.45E-06	Adj.R.Squared	0.0166
LM.Cannabis_x_Herb.THC_x_Daily.Interpol.	1.1 (0.5, 1.7)	0.0004	Statistic	15.0685
LM.Cannabis_x_Resin.THC_x_Daily.Interpol.	-1.31 (-2, -0.62)	0.0003	Deg.Freedom	6,115
Herb	8.41 (4.06, 12.76)	0.0002	P-Value	1.15E-12
Cocaine	-0.38 (-0.7, -0.05)	0.0263		
Income	0 (0, 0)	7.45E-05		
**Interactive**				
**Rate ~ Tobacco + Daily.Interpol. * LM.Cannabis_x_Herb.THC_x_Daily.Interpol. + LM.Cannabis_x_Resin.THC_x_Daily.Interpol. + LM.Cannabis_x_Resin.THC + Alcohol + Amphetamines + Cocaine + Income**				
Daily.Interpol.	149 (50.22, 247.78)	0.0038	Adj.R.Squared	0.2309
Resin	3.51 (1.14, 5.88)	0.0046	Statistic	15.2721
Herb	15.2 (9.87, 20.53)	1.75E-07	Deg.Freedom	10,111
LM.Cannabis_x_Herb.THC_x_Daily.Interpol.	1.29 (0.43, 2.15)	0.0040	P-Value	<2.2E-16
LM.Cannabis_x_Resin.THC_x_Daily.Interpol.	-1.52 (-2.52, -0.52)	0.0036		
Amphetamines	0.26 (0.07, 0.45)	0.0074		
Cocaine	-0.38 (-0.69, -0.07)	0.0162		
Income	0 (0, 0)	0.0009		
Tobacco: Daily.Interpol.	-3.89 (-7.61, -0.17)	0.0427		

Resin: Herb	-48.8 (-68.6, -29)	4.39E-06		
**2 Lags**				
**Rate ~ Tobacco + Daily.Interpol. * LM.Cannabis_x_Resin.THC_x_Daily.Interpol. + LM.Cannabis_x_Herb.THC_x_Daily.Interpol. + LM.Cannabis_x_Herb.THC + Alcohol + Daily.Interpol. + Amphetamines + Cocaine + Income**				
Herb	15.69 (8.88, 22.49)	1.87E-05	Adj.R.Squared	0.0660
Resin: Herb	-38.35 (-59.09, -17.61)	0.0005	Statistic	11.5482
			Deg.Freedom	2,91
			P-Value	3.39E-05

Table Notes: L.M. – Last Month

Supplementary Table S14.: Panel Regression Results for Congenital Posterior Urethral Valve

Parameters			Model Parameters	
**Term**	**Estimate (C.I.)**	**P-Value**	**Parameter**	**Value**
**Additive**				
**Rate ~ Tobacco + Alcohol + LM.Cannabis_x_Herb.THC_x_Daily.Interpol. + LM.Cannabis_x_Resin.THC_x_Daily.Interpol. + LM.Cannabis_x_Resin.THC + Daily.Interpol. + Resin + Herb + Amphetamines + Cocaine + Income**				
Alcohol	0.09 (0.01, 0.16)	0.0309	Adj.R.Squared	0.0426
LM.Cannabis_x_Herb.THC_x_Daily.Interpol.	-1.13 (-1.53, -0.73)	2.18E-07	Statistic	7.5836
LM.Cannabis_x_Resin.THC_x_Daily.Interpol.	1.25 (0.81, 1.69)	2.25E-07	Deg.Freedom	6,115
Daily.Interpol.	-61.5 (-81.69, -41.31)	2.92E-08	P-Value	7.21E-07
Cocaine	0.69 (0.39, 0.99)	1.40E-05		
Income	0 (0, 0)	0.0053		
**Interactive**				
**Rate ~ Tobacco + Daily.Interpol. * LM.Cannabis_x_Herb.THC_x_Daily.Interpol. + LM.Cannabis_x_Resin.THC_x_Daily.Interpol. + LM.Cannabis_x_Resin.THC + Alcohol + Amphetamines + Cocaine + Income**				
LM.Cannabis_x_Resin.THC_x_Daily.Interpol.	0.34 (0.21, 0.48)	3.38E-06	Adj.R.Squared	0.0934
LM.Cannabis_x_Resin.THC	2.16 (1.54, 2.77)	2.66E-10	Statistic	15.0969
Alcohol	0.09 (0.04, 0.14)	0.0005	Deg.Freedom	4,117
Daily.Interpol.: LM.Cannabis_x_Herb.THC_x_Daily.Interpol.	-96.23 (-121.24, -71.22)	1.09E-11	P-Value	5.58E-10
**2 Lags**				
**Rate ~ Tobacco + Daily.Interpol. * LM.Cannabis_x_Resin.THC_x_Daily.Interpol. + LM.Cannabis_x_Herb.THC_x_Daily.Interpol. + LM.Cannabis_x_Herb.THC + Alcohol + Daily.Interpol. + Amphetamines + Cocaine + Income**				
Daily.Interpol.	-18.48 (-28.79, -8.18)	0.0007	Adj.R.Squared	-0.0099
Alcohol	0.11 (0.05, 0.18)	0.0014	Statistic	5.0773
Daily.Interpol.: LM.Cannabis_x_Resin.THC_x_Daily.Interpol.	9.01 (0.2, 17.81)	0.0481	Deg.Freedom	3,90

			P-Value	0.0027

Table Notes: L.M. – Last Month

Supplementary Figure

Edited (Red) Links (Blue) to European Country Neighbourhood Links for Uro-Nephrological Anomalies Dataset

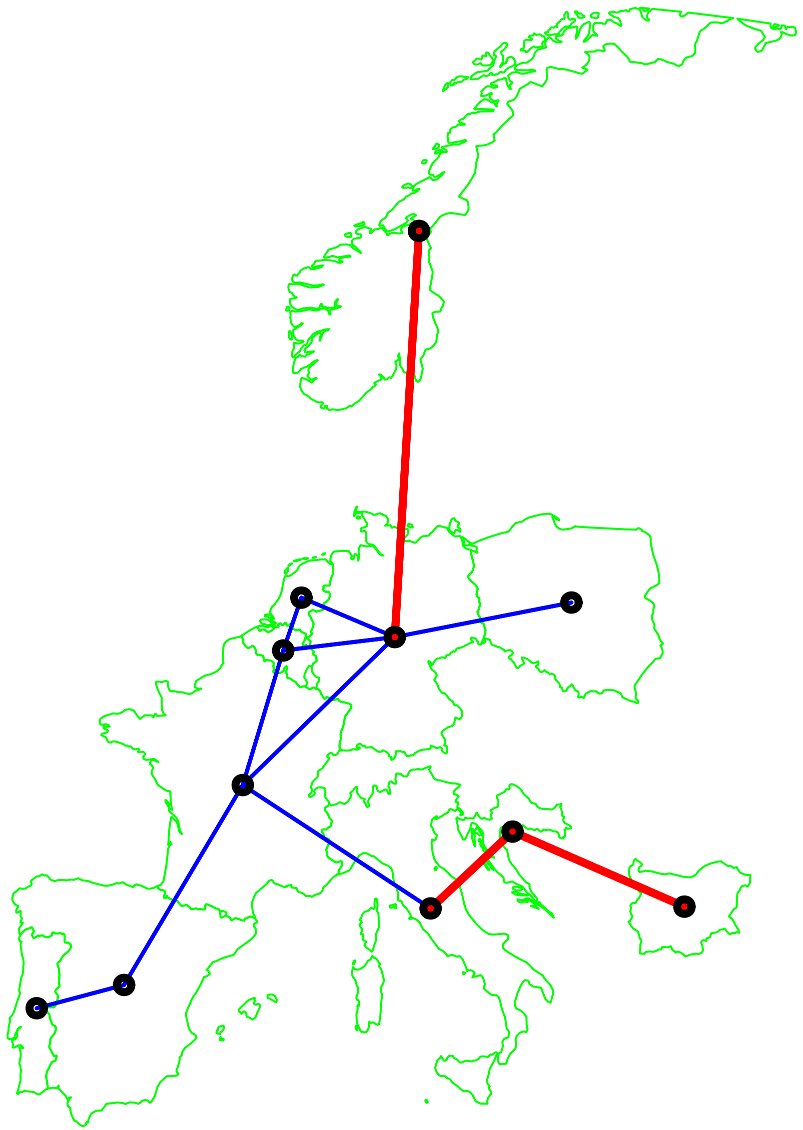

Final Country Neighbourhood Links for European Uro-Nephrological Anomalies Dataset

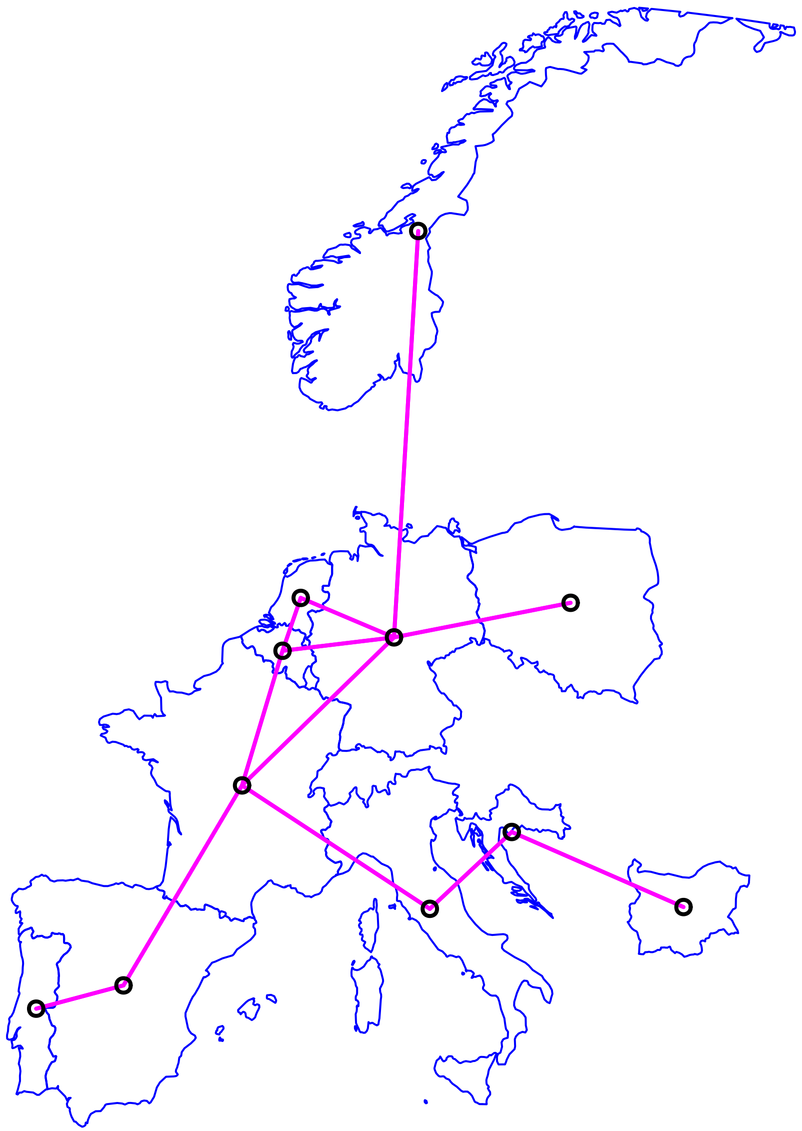

Figure Legend for Supplementary Figure S1.

Interstate geospatial links. (A) Raw and edited interstate links and (B) final interstate links used for the spatial weights matrix.
